# Whole egg consumption increases gene expression within the glutathione pathway in the liver of Zucker Diabetic Fatty rats

**DOI:** 10.1371/journal.pone.0240885

**Published:** 2020-11-03

**Authors:** Joe L. Webb, Amanda E. Bries, Brooke Vogel, Claudia Carrillo, Lily Harvison, Timothy A. Day, Michael J. Kimber, Rudy J. Valentine, Matthew J. Rowling, Stephanie Clark, Elizabeth M. McNeill, Kevin L. Schalinske

**Affiliations:** 1 Department of Food Science and Human Nutrition, Iowa State University, Ames, IA, United States of America; 2 Interdepartmental Graduate Program in Nutritional Sciences, Iowa State University, Ames, IA, United States of America; 3 Department of Biomedical Sciences, Iowa State University College of Veterinary Medicine, Ames, IA, United States of America; 4 Department of Kinesiology, Iowa State University, Ames, IA, United States of America; 5 Genetics and Genomics Graduate Program, Iowa State University, Ames, IA, United States of America; Universidade do Estado do Rio de Janeiro, BRAZIL

## Abstract

Nutrigenomic evidence supports the idea that Type 2 Diabetes Mellitus (T2DM) arises due to the interactions between the transcriptome, individual genetic profiles, lifestyle, and diet. Since eggs are a nutrient dense food containing bioactive ingredients that modify gene expression, our goal was to examine the role of whole egg consumption on the transcriptome during T2DM. We analyzed whether whole egg consumption in Zucker Diabetic Fatty (ZDF) rats alters microRNA and mRNA expression across the adipose, liver, kidney, and prefrontal cortex tissue. Male ZDF (fa/fa) rats (*n* = 12) and their lean controls (fa/+) (*n* = 12) were obtained at 6 wk of age. Rats had *ad libitum* access to water and were randomly assigned to a modified semi-purified AIN93G casein-based diet or a whole egg-based diet, both providing 20% protein (w/w). TotalRNA libraries were prepared using QuantSeq 3' mRNA-Seq and Lexogen smallRNA library prep kits and were further sequenced on an Illumina HighSeq3000. Differential gene expression was conducted using DESeq2 in R and Benjamini-Hochberg adjusted *P*-values controlling for false discovery rate at 5%. We identified 9 microRNAs and 583 genes that were differentially expressed in response to 8 wk of consuming whole egg-based diets. Kyto Encyclopedia of Genes and Genomes/Gene ontology pathway analyses demonstrated that 12 genes in the glutathione metabolism pathway were upregulated in the liver and kidney of ZDF rats fed whole egg. Whole egg consumption primarily altered glutathione pathways such as conjugation, methylation, glucuronidation, and detoxification of reactive oxygen species. These pathways are often negatively affected during T2DM, therefore this data provides unique insight into the nutrigenomic response of dietary whole egg consumption during the progression of T2DM.

## Introduction

Type 2 Diabetes Mellitus (T2DM) is an insulin independent metabolic disease characterized by chronic hyperglycemia and concomitant insulin resistance and it is estimated that greater than 415 million adults worldwide have T2DM [1]. Oxidative stress is a potential key mediator in the pathogenesis of T2DM and may underlie the progressive development of hyperglycemia and insulin resistance [2]. More specifically, reports demonstrate that glutathione (a major intracellular antioxidant) enzymes are diminished in the liver and brain of T2DM animal models [3]. Sekhar and colleagues examined the ability of patients with uncontrolled and controlled T2DM to synthesize glutathione via measuring isotopically labelled glycine [4]. They reported that patients with uncontrolled T2DM were severely deficient in the ability to maintain glutathione metabolism in cardiac tissue [5], which may be, in part, due to hyperglycemia decreasing L-cysteine concentrations [6] and the reduced flux of methionine to cysteine [7]. Because of the deleterious effects of hyperglycemia on organ function, it is important to consider the global transcriptomic effects of T2DM. Similar to humans, the Zucker Diabetic Fatty (ZDF) rat model of T2DM also displays increased oxidative stress [8], whereby endogenous protective antioxidants like glutathione are similarly downregulated in ZDF rats [9]. The gene expression profiles in animal models of T2DM, such as the ZDF rat, is consistent with gene expression profiles of humans with T2DM [8], making this a suitable model to explore the global gene expression effects of diet in the ZDF rat.

Dietary treatments with bioactive foods such as cocoa or Shenyuan granules [9, 10] in ZDF rats have been shown to reduce oxidative stress or attenuate renal injury in the presence of T2DM-related nephropathy [11]. Consumption of eggs as a bioactive food during T2DM in humans remains controversial [12–15], but eggs have been shown to display antioxidative properties, which may be beneficial during the progression of T2DM [16]. Additionally, our laboratory has consistently reported that long-term whole egg (WE) consumption improves metabolic parameters during T2DM such as the maintenance of circulating vitamin D concentrations, decreased weight gain, and nephroprotection via reduced proteinuria in male ZDF rats [17, 18]. These are important findings, as vitamin D deficiency, increased adiposity, and kidney failure have collectively been suggested to exacerbate oxidative stress during T2DM [19].

While the literature surrounding the effects of dietary WE on insulin resistance during T2DM is inconclusive in both rodent [20] and human population studies [21], there are no studies to date examining the molecular mechanisms underlying how WE consumption affects the transcriptome across multiple tissues. Longitudinal, prospective, and comprehensive meta-analyses have been performed to assess the independent risk factors of increased dietary egg consumption on chronic diseases [22, 23]. Because of the highly controversial science of whole egg consumption on increased cardiovascular disease in patients with T2DM, it is important to examine the possible underlying molecular targets and drivers of whole egg consumption on disease. Ultimately, analyzing the transcriptomic impact of egg consumption would provide us with a better understanding of the nutrigenomic actions that dietary egg consumption contributes to T2DM, and bridge the gap in our understanding of how whole eggs may affect the physiological progression of T2DM. Therefore, the objective of this study was to determine the influence of WE consumption on gene and microRNA expression profiles in a ZDF rat model of progressive T2DM. We examined the transcriptomes from the adipose, liver, kidney, and prefrontal cortex (PFC) tissues to determine how WE consumption alters gene expression and examined whether these changes correspond to altered microRNA expression profiles in T2DM.

## Results and discussion

Whole eggs have predominantly been criticized for their associated risk of developing chronic diseases [24], yet the benefits of WE consumption have also been reported [13]. For instance, several groups have suggested that WE provide antioxidant properties [13, 25], either through antioxidant peptides in the egg yolk [11] or other reactive oxygen species-reducing nutrients [26]. Other studies examining the role of quail egg consumption in rat models of T2DM have demonstrated upregulation of glutathione metabolism in alloxan-induced T2DM in Wistar rats [25] and improved oxidative stress profiles in streptozotocin-injected rats [27]. Raza and colleagues [27] identified that in diabetic rat liver glutathione content and glutathione S-transferase (GST) activity were decreased 65% while also observing that brain glutathione and GST activity were increased two-fold as a result of a T2DM phenotype.

### Total RNASeq differential expression

When comparing the WE versus casein (CAS) in ZDF rats and their lean controls, differential expression analyses of the mRNAseq data resulted in 583 differentially expressed genes (DEGs) across four tissues in both genotypes **(Table 1)**. **S1 Table** contains the results from DESeq2 with the results for each gene across all four tissues with data on individual genes. **S2 Table** contains raw mRNA read counts for each tissue and rat across both genotypes. Among the lean controls, 13 genes were differentially expressed in the adipose tissue, 32 in the liver, and 6 in the kidney. Notably, none of the genes were differentially expressed in the PFC between dietary treatments in the lean rats. In the ZDF rats, dietary WE consumption resulted in 532 total DEGs across all tissues where 50 genes were differentially expressed in adipose tissue, 474 in the liver, 6 in the kidney and 2 genes in the PFC following multiple testing correction using the false discovery rate (FDR) threshold of 5%. We demonstrated that consuming WE-based diets for 8 wk resulted in significant alterations in oxidative stress pathways, as well as glutathione metabolism pathways. While there were tissue-specific changes in gene expression, glutathione metabolism was altered in the kidney and liver among ZDF rats, and in the kidney of lean controls were significantly upregulated. Overall, these data highlight how consumption of WE-based diets can provide beneficial effects through modifying gene expression of oxidative reduction targets.

**Table 1:** Differentially expressed genes in the liver, kidney, adipose and PFC tissues stratified according to each genotype^1^^-^^3^.

**Genotype**	**Tissue**	**Ensembl_ID (ENSRNO)**	**Symbol**	**Gene Name**	**L2FC**	**P-value**^**3**^
ZDF	Adipose Downregulated	G00000011039	Gch1	GTP cyclohydrolase 1	-2.71	2.1E-05
		G00000040108	RGD1565355	similar to fatty acid translocase/CD36	-2.65	2.7E-07
		G00000011024	Zdhhc20	zinc finger DHHC-type palmitoyltransferase 20	-2.49	1.2E-04
		G00000006946	Arhgap9	Rho GTPase activating protein 9	-2.49	2.6E-06
		G00000006715	Ccr1	C-C motif chemokine receptor 1	-2.39	7.4E-06
		G00000032546	Dot1l	DOT1 like histone lysine methyltransferase	-2.12	1.9E-06
		G00000034230	Fcrl1	Fc receptor-like 1	-2.09	6.9E-05
		G00000019283	P2ry2	purinergic receptor P2Y2	-2.07	1.2E-04
		G00000022975	Nfam1	NFAT activating protein with ITAM motif 1	-1.92	3.1E-04
		G00000013917	Igsf10	immunoglobulin superfamily, member 10	-1.91	2.4E-05
		G00000049115	Ccr5	C-C motif chemokine receptor 5	-1.89	4.3E-07
		G00000015895	B4galt6	beta-1,4-galactosyltransferase 6	-1.84	5.1E-05
		G00000020479	Pik3c2a	phosphatidylinositol-4-phosphate 3-kinase, catalytic subunit type 2 alpha	-1.83	2.2E-04
		G00000061379	C7	complement C7	-1.82	1.8E-04
		G00000011927	Sdc3	syndecan 3	-1.82	2.3E-04
		G00000026644	Glipr1	GLI pathogenesis-related 1	-1.77	6.4E-05
		G00000011946	Ptn	pleiotrophin	-1.74	4.8E-05
		G00000013922	Dok2	docking protein 2	-1.61	2.7E-04
		G00000013526	Rassf4	Ras association domain family member 4	-1.60	3.1E-06
		G00000001989	Alcam	activated leukocyte cell adhesion molecule	-1.57	1.5E-05
		G00000016643	Lpcat2	lysophosphatidylcholine acyltransferase 2	-1.52	2.9E-04
		G00000003835	Slc43a2	solute carrier family 43 member 2	-1.52	1.6E-04
		G00000019077	Lipa	lipase A, lysosomal acid type	-1.51	3.8E-05
		G00000009347	Arhgap25	Rho GTPase activating protein 25	-1.49	2.1E-04
		G00000000257	Smpd3	sphingomyelin phosphodiesterase 3	-1.47	2.8E-04
		G00000012616	Ppt1	palmitoyl-protein thioesterase 1	-1.41	2.7E-04
		G00000009331	Hck	HCK proto-oncogene, Src family tyrosine kinase	-1.30	4.6E-05
		G00000010183	Gask1b	golgi associated kinase 1B	-1.26	2.7E-04
		G00000017022	Cerk	ceramide kinase	-1.25	3.2E-04
		G00000008465	Tmem176b	transmembrane protein 176B	-1.23	2.4E-04
		G00000010208	Timp1	TIMP metallopeptidase inhibitor 1	-1.20	1.8E-04
**ZDF**	**Adipose Upregulated**	**Ensembl_ID (ENSRNO)**	**Gene Symbol**	**Gene Name**	**L2FC**	**P-value**
		G00000015072	Ptgr1	prostaglandin reductase 1	1.15	2.2E-04
		G00000010389	Ndrg2	NDRG family member 2	1.30	2.0E-04
		G00000037446	Pxmp2	peroxisomal membrane protein 2	1.30	2.3E-04
		G00000002896	Prdx6	peroxiredoxin 6	1.37	3.1E-04
		G00000019328	Phgdh	phosphoglycerate dehydrogenase	1.45	3.4E-04
		G00000021316	Tmem98	transmembrane protein 98	1.48	2.1E-04
		G00000046858	MGC109340	similar to Microsomal signal peptidase 23 kDa subunit (SPase 22 kDa subunit) (SPC22/23)	1.52	9.7E-05
		G00000017012	Coq7	coenzyme Q7, hydroxylase	1.56	4.5E-05
		G00000021524	Mrap	melanocortin 2 receptor accessory protein	1.69	1.6E-04
		G00000017226	Slc2a4	solute carrier family 2 member 4	1.87	1.8E-04
		G00000002579	Parm1	prostate androgen-regulated mucin-like protein 1	1.89	7.0E-06
		G00000001001	Retn	resistin	1.95	3.0E-05
		G00000008615	Mal2	mal, T-cell differentiation protein 2	2.09	1.6E-04
		G00000019412	Rhbg	Rh family B glycoprotein	2.12	1.3E-05
		G00000009715	Me1	malic enzyme 1	2.13	2.0E-06
		G00000012404	Thrsp	thyroid hormone responsive	2.43	2.4E-06
		G00000019914	Tlcd3b	TLC domain containing 3B	2.47	4.8E-10
		G00000045636	Fasn	fatty acid synthase	3.25	1.7E-11
		G00000049911	LOC102556347	carbonyl reductase [NADPH] 1-like	4.33	6.0E-18
**Lean**	**Adipose Downregulated**	**Ensembl_ID (ENSRNO)**	**Gene Symbol**	**Gene Name**	**L2FC**	**P-value**
		G00000016700	Tcf21	transcription factor 21	-2.82	3.2E-06
**Lean**	**Adipose Upregulated**	**Ensembl_ID (ENSRNO)**	**Gene Symbol**	**Gene Name**	**L2FC**	**P-value**
		G00000013733	Ppp4r1	protein phosphatase 4, regulatory subunit 1	2.68	2.4E-05
		G00000009536	Pgp	phosphoglycolate phosphatase	2.69	3.4E-05
		G00000031789	Rangap1	RAN GTPase activating protein 1	2.97	3.9E-05
		G00000005082	Irf6	interferon regulatory factor 6	3.25	1.3E-05
		G00000031934	Enah	ENAH, actin regulator	3.26	2.4E-05
		G00000011296	Cenpn	centromere protein N	3.34	2.8E-05
		G00000056550	Epb41l4b	erythrocyte membrane protein band 4.1 like 4B	3.51	1.9E-05
		G00000021589	Nexmif	neurite extension and migration factor	4.43	2.9E-05
		G00000008713	Slc41a2	solute carrier family 41 member 2	4.51	4.8E-07
		G00000033262	Reep6	receptor accessory protein 6	4.72	1.0E-05
		G00000003098	Prom1	prominin 1	5.25	2.6E-06
		G00000015403	Cd52	CD52 molecule	7.66	2.5E-07
**ZDF**	**PFC Downregulated**	**Ensembl_ID (ENSRNO)**	**Gene Symbol**	**Gene Name**	**L2FC**	**P-value**
		G00000033932	AY172581.22–201	AY172581.22–201	-5.21	1.1E-05
		G00000032112	AY172581.14	AY172581.14	-4.73	1.5E-05
**ZDF**	**PFC Upregulated**	**Ensembl_ID (ENSRNO)**	**Gene Symbol**	**Gene Name**	**L2FC**	**P-value**
		**None**				
**Lean**	**PFC Downregulated**	**Ensembl_ID (ENSRNO)**	**Gene Symbol**	**Gene Name**	**L2FC**	**P-value**
		**None**				
**Lean**	**PFC Upregulated**	**Ensembl_ID (ENSRNO)**	**Gene Symbol**	**Gene Name**	**L2FC**	**P-value**
		**None**				
**ZDF**	**Kidney Downregulated**	**Ensembl_ID (ENSRNO)**	**Gene Symbol**	**Gene Name**	**L2FC**	**P-value**
		G00000020204	Srp19	signal recognition particle 19	-1.72	3.3E-05
		G00000004794	Rtn1	reticulon 1	-2.01	5.1E-05
		G00000055471	Ywhah	tyrosine 3-monooxygenase/tryptophan 5-monooxygenase activation protein, eta	-1.29	5.5E-05
		G00000003357	Col3a1	collagen type III alpha 1 chain	-1.46	6.4E-05
**ZDF**	**Kidney Upregulated**	**Ensembl_ID (ENSRNO)**	**Gene Symbol**	**Gene Name**	**L2FC**	**P-value**
		G00000018237	Gstp1	glutathione S-transferase pi 1	2.04	5.2E-07
		G00000018940	CNT1	solute carrier family 28 member 1	1.65	7.8E-05
**Lean**	**Kidney Downregulated**	**Ensembl_ID (ENSRNO)**	**Gene Symbol**	**Gene Name**	**L2FC**	**P-value**
		G00000020151	Cdh1	cadherin 1	-1.08	1.4E-05
		G00000013062	Cyp24a1	cytochrome P450, family 24, subfamily a, polypeptide 1	-1.30	4.3E-06
		G00000012956	Tgm2	transglutaminase 2	-1.34	3.5E-05
		G00000004019	Phlda1	pleckstrin homology-like domain, family A, member 1	-2.30	2.7E-08
**Lean**	**Kidney Upregulated**	**Ensembl_ID (ENSRNO)**	**Gene Symbol**	**Gene Name**	**L2FC**	**P-value**
		G00000029726	Gstm1	glutathione S-transferase mu 1	1.40	1.5E-05
		G00000053811	Arg2	arginase 2	1.51	3.5E-05
		G00000000576	Anapc16	anaphase promoting complex subunit 16	2.11	3.3E-05
**ZDF**	**Liver Downregulated**	**Ensembl_ID (ENSRNO)**	**Gene Symbol**	**Gene Name**	**L2FC**	**P-value**
		G00000014320	Inhba	inhibin subunit beta A	-4.77	3.2E-12
		G00000007923	Cgref1	cell growth regulator with EF hand domain 1	-3.74	4.4E-09
		G00000004307	Tor3a	torsin family 3	-3.55	6.1E-18
		G00000034190	Ighm	immunoglobulin heavy constant mu	-3.38	1.4E-16
		G00000003802	Pttg1	PTTG1 regulator of sister chromatid separation	-3.35	1.2E-05
		G00000007060	Plin2	perilipin 2	-3.31	6.8E-28
		G00000045636	Fasn	fatty acid synthase	-3.31	1.5E-10
		G00000022256	Cxcl10	C-X-C motif chemokine ligand 10	-3.30	4.4E-04
		G00000009019	Slc6a6	solute carrier family 6 member 6	-3.09	6.0E-11
		G00000025691	Pla2g7	phospholipase A2 group VII	-3.03	4.5E-06
		G00000020035	Cyp17a1	cytochrome P450	-3.00	2.1E-09
		G00000020480	Fads1	fatty acid desaturase 1	-2.93	1.5E-13
		G00000000658	Acacb	acetyl-CoA carboxylase beta	-2.91	4.0E-18
		G00000030154	Cyp4a2	cytochrome P450	-2.88	5.0E-04
		G00000021802	Isg15	ISG15 ubiquitin-like modifier	-2.72	2.4E-03
		G00000001052	Slc25a30	solute carrier family 25	-2.58	1.8E-09
		G00000001963	Mx2	MX dynamin like GTPase 2	-2.56	2.1E-03
		G00000040151	Sdr16c6	short chain dehydrogenase/reductase family 16C	-2.55	6.3E-04
		G00000017914	Cavin3	caveolae associated protein 3	-2.51	1.4E-14
		G00000006859	Insig1	insulin induced gene 1	-2.50	3.4E-13
		G00000006204	Slc30a3	solute carrier family 30 member 3	-2.47	3.0E-07
		G00000016353	Nim1k	NIM1 serine/threonine protein kinase	-2.40	3.6E-08
		G00000016011	Plekhg1	pleckstrin homology and RhoGEF domain containing G1	-2.40	7.8E-05
		G00000028137	Mki67	marker of proliferation Ki-67	-2.38	1.5E-04
		G00000014476	Evl	Enah/Vasp-like	-2.37	3.4E-04
		G00000008022	Apaf1	apoptotic peptidase activating factor 1	-2.36	7.1E-05
		G00000053891	Phf11	PHD finger protein 11	-2.34	6.4E-08
		G00000010819	Hspa4l	heat shock protein family A (Hsp70) member 4 like	-2.32	6.9E-06
		G00000021150	Plcb3	phospholipase C beta 3	-2.31	3.1E-05
		G00000001414	Serpine1	serpin family E member 1	-2.27	1.2E-04
		G00000016924	Acly	ATP citrate lyase	-2.25	5.5E-17
		G00000045560	Gvin1	GTPase	-2.25	2.1E-06
		G00000020503	Cbln3	cerebellin 3 precursor	-2.22	1.4E-06
		G00000052444	Samd9	sterile alpha motif domain containing 9	-2.22	3.3E-04
		G00000005209	Spred1	sprouty-related	-2.21	1.7E-05
		G00000010888	Ankrd33b	ankyrin repeat domain 33B	-2.20	2.1E-06
		G00000047218	Clic5	chloride intracellular channel 5	-2.20	1.7E-03
		G00000009481	Ddhd1	DDHD domain containing 1	-2.19	2.0E-04
		G00000022242	Cxcl9	C-X-C motif chemokine ligand 9	-2.16	1.3E-05
		G00000008807	Rp1	RP1	-2.08	4.7E-05
		G00000014426	Lox	lysyl oxidase	-2.07	1.9E-03
		G00000015498	Il17rb	interleukin 17 receptor B	-2.07	2.3E-04
		G00000051965	Smad4	SMAD family member 4	-2.07	8.7E-04
		G00000017512	Aldh3b1	aldehyde dehydrogenase 3 family	-2.05	1.0E-04
		G00000057092	Slfn4	schlafen family member 4	-2.05	6.2E-06
		G00000012685	Adck1	aarF domain containing kinase 1	-2.04	1.8E-03
		G00000011268	Chd5	chromodomain helicase DNA binding protein 5	-2.02	2.3E-03
		G00000032374	Paqr9	progestin and adipoQ receptor family member 9	-2.01	3.3E-14
		G00000020272	Elapor1	endosome-lysosome associated apoptosis and autophagy regulator 1	-1.97	1.0E-04
		G00000061118	LOC102551095	uncharacterized LOC102551095	-1.96	9.6E-05
		G00000061527	Gck	glucokinase	-1.93	4.4E-07
		G00000053460	Acot3	acyl-CoA thioesterase 3	-1.91	1.4E-04
		G00000005043	Cpeb2	cytoplasmic polyadenylation element binding protein 2	-1.91	2.0E-03
		G00000017332	Dapk2	death-associated protein kinase 2	-1.87	3.8E-04
		G00000034013	Acaca	acetyl-CoA carboxylase alpha	-1.86	4.5E-05
		G00000017611	Tnp1	transition protein 1	-1.86	2.0E-03
		G00000012603	Sestd1	SEC14 and spectrin domain containing 1	-1.85	1.2E-03
		G00000025558	Palm2	paralemmin 2	-1.84	5.7E-06
		G00000018461	Pdgfrb	platelet derived growth factor receptor beta	-1.82	1.0E-03
		G00000016123	Rnf144b	ring finger protein 144B	-1.80	5.5E-17
		G00000013111	Mettl3	methyltransferase-like 3	-1.78	6.7E-04
		G00000045679	Apoa1	apolipoprotein A1	-1.78	1.1E-11
		G00000001926	Cldn1	claudin 1	-1.78	1.8E-06
		G00000005600	Nr4a2	nuclear receptor subfamily 4	-1.77	4.2E-04
		G00000012148	Trio	trio Rho guanine nucleotide exchange factor	-1.76	7.0E-04
		G00000004626	Slc34a2	solute carrier family 34 member 2	-1.76	8.7E-05
		G00000009360	Sh3bp1	SH3-domain binding protein 1	-1.74	2.2E-03
		G00000010890	Bmp1	bone morphogenetic protein 1	-1.71	1.8E-07
		G00000011820	Acp3	acid phosphatase 3	-1.69	1.1E-04
		G00000007591	Slc45a3	solute carrier family 45	-1.68	8.9E-05
		G00000006170	Bach2	BTB domain and CNC homolog 2	-1.68	1.2E-03
		G00000028895	Rtp4	receptor (chemosensory) transporter protein 4	-1.66	5.3E-05
		G00000002773	Rgs4	regulator of G-protein signaling 4	-1.65	5.0E-04
		G00000007234	Cyp51	cytochrome P450	-1.64	1.2E-09
		G00000020918	Ccnd1	cyclin D1	-1.64	7.0E-09
		G00000028941	Zbed3	zinc finger	-1.63	8.4E-06
		G00000012681	Lgals9	galectin 9	-1.63	2.8E-13
		G00000001640	Tomm70	translocase of outer mitochondrial membrane 70	-1.63	2.6E-03
		G00000009117	Otub2	OTU deubiquitinase	-1.62	1.9E-04
		G00000005726	Pclo	piccolo (presynaptic cytomatrix protein)	-1.62	6.2E-04
		G00000051171	G6pc	glucose-6-phosphatase	-1.61	1.6E-04
		G00000016552	Hmgcs1	3-hydroxy-3-methylglutaryl-CoA synthase 1	-1.60	4.4E-15
		G00000004577	Fez2	fasciculation and elongation protein zeta 2	-1.60	1.9E-04
		G00000000547	Tspyl4	TSPY-like 4	-1.59	5.0E-04
		G00000017120	Abhd2	abhydrolase domain containing 2	-1.59	1.9E-07
		G00000015906	Tgif1	TGFB-induced factor homeobox 1	-1.56	1.1E-03
		G00000008144	Irf1	interferon regulatory factor 1	-1.54	2.4E-06
		G00000007319	Trib3	tribbles pseudokinase 3	-1.54	8.5E-06
		G00000018467	Mitd1	microtubule interacting and trafficking domain containing 1	-1.52	1.9E-03
		G00000023238	Dgkd	diacylglycerol kinase	-1.52	1.2E-05
		G00000020776	Dhcr7	7-dehydrocholesterol reductase	-1.51	1.1E-05
		G00000033824	Gpd2	glycerol-3-phosphate dehydrogenase 2	-1.51	5.5E-04
		G00000003442	Adora1	adenosine A1 receptor	-1.50	2.3E-06
		G00000025689	Abhd1	abhydrolase domain containing 1	-1.50	2.1E-07
		G00000018198	Dapk1	death associated protein kinase 1	-1.48	5.3E-04
		G00000029668	Wfdc21	WAP four-disulfide core domain 21	-1.45	1.6E-04
		G00000006280	Pcsk9	proprotein convertase subtilisin/kexin type 9	-1.44	4.7E-07
		G00000008215	Trim47	tripartite motif-containing 47	-1.44	1.3E-04
		G00000014013	Map4k4	mitogen-activated protein kinase kinase kinase kinase 4	-1.43	1.1E-05
		G00000018627	Plekhb1	pleckstrin homology domain containing B1	-1.43	8.9E-05
		G00000007713	Tmcc3	transmembrane and coiled-coil domain family 3	-1.43	2.6E-05
		G00000000987	Ptcd1	pentatricopeptide repeat domain 1	-1.43	1.0E-03
		G00000014702	Elovl2	ELOVL fatty acid elongase 2	-1.43	2.8E-11
		G00000037595	Gpbp1l1	GC-rich promoter binding protein 1-like 1	-1.41	2.3E-04
		G00000001205	Agpat3	1-acylglycerol-3-phosphate O-acyltransferase 3	-1.41	3.8E-09
		G00000019776	Sh3gl3	SH3 domain containing GRB2 like 3	-1.40	4.1E-05
		G00000012622	Mmp15	matrix metallopeptidase 15	-1.40	1.9E-03
		G00000023856	Agxt	alanine—glyoxylate and serine—pyruvate aminotransferase	-1.39	7.5E-11
		G00000042560	Bag4	BCL2-associated athanogene 4	-1.38	1.2E-03
		G00000013841	Dcaf1	DDB1 and CUL4 associated factor 1	-1.38	2.1E-03
		G00000019995	Dnajc18	DnaJ heat shock protein family (Hsp40) member C18	-1.37	2.4E-04
		G00000019996	Slc16a1	solute carrier family 16 member 1	-1.36	1.6E-04
		G00000023226	S100a10	S100 calcium binding protein A10	-1.35	2.2E-04
		G00000006227	Ifih1	interferon induced with helicase C domain 1	-1.35	7.5E-04
		G00000019005	Pde8a	phosphodiesterase 8A	-1.34	1.5E-03
		G00000021259	Prnp	prion protein	-1.33	1.7E-03
		G00000055909	Apoa4	apolipoprotein A4	-1.33	2.6E-10
		G00000008012	Abcb4	ATP binding cassette subfamily B member 4	-1.33	4.1E-10
		G00000021405	Cyp2c7	cytochrome P450	-1.32	1.0E-08
		G00000012181	Lpl	lipoprotein lipase	-1.31	5.3E-05
		G00000056041		AABR07062570	-1.30	2.5E-03
		G00000016815	Tmem135	transmembrane protein 135	-1.29	4.1E-05
		G00000019422	Egr1	early growth response 1	-1.29	1.1E-05
		G00000054077		Aabr07024870	-1.29	1.4E-03
		G00000008194	Znfx1	zinc finger	-1.28	5.5E-04
		G00000009076	Ttpal	alpha tocopherol transfer protein like	-1.27	1.7E-03
		G00000005825	Lyz2	lysozyme 2	-1.26	2.7E-05
		G00000032293	Polg	DNA polymerase gamma	-1.26	2.9E-04
		G00000008586	Aldh1l2	aldehyde dehydrogenase 1 family	-1.26	3.5E-04
		G00000010805	Fabp4	fatty acid binding protein 4	-1.25	2.5E-03
		G00000016044	Mab21l3	mab-21 like 3	-1.25	1.0E-04
		G00000000459	Psmb9	proteasome 20S subunit beta 9	-1.25	7.4E-04
		G00000015124	Gpam	glycerol-3-phosphate acyltransferase	-1.25	1.4E-03
		G00000020871	Ltbp4	latent transforming growth factor beta binding protein 4	-1.22	2.1E-03
		G00000016516	Mbp	myelin basic protein	-1.22	1.2E-04
		G00000007324	Plxna2	plexin A2	-1.22	2.4E-07
		G00000001821	Adipoq	adiponectin	-1.21	2.7E-03
		G00000020573	Efna1	ephrin A1	-1.19	1.7E-04
		G00000004606	Meis1	Meis homeobox 1	-1.19	8.3E-04
		G00000001647	Ets2	ETS proto-oncogene 2	-1.17	9.1E-09
		G00000059043	Itch	itchy E3 ubiquitin protein ligase	-1.17	1.4E-03
		G00000006787	Dhcr24	24-dehydrocholesterol reductase	-1.16	1.9E-12
		G00000015121	N4bp1	Nedd4 binding protein 1	-1.15	3.0E-04
		G00000042771	Apol3	apolipoprotein L	-1.14	7.6E-08
		G00000023664	Lepr	leptin receptor	-1.12	2.5E-03
		G00000000451	RT1-Ba	RT1 class II	-1.12	1.1E-03
		G00000012782	Cemip2	cell migration inducing hyaluronidase 2	-1.11	1.0E-05
		G00000014766	Galt	galactose-1-phosphate uridylyltransferase	-1.11	1.7E-05
		G00000014718	Acsl3	acyl-CoA synthetase long-chain family member 3	-1.11	1.4E-03
		G00000017428	Map1b	microtubule-associated protein 1B	-1.10	1.7E-03
		G00000018517	Trim21	tripartite motif-containing 21	-1.09	2.0E-03
		G00000001426	Prkrip1	PRKR interacting protein 1	-1.09	2.2E-03
		G00000028448	Elovl1	ELOVL fatty acid elongase 1	-1.08	2.2E-03
		G00000005695	Mgp	matrix Gla protein	-1.07	1.2E-03
		G00000017558	Tubb2a	tubulin	-1.07	7.2E-04
		G00000012876	Slc6a13	solute carrier family 6 member 13	-1.07	7.1E-05
		G00000018960	Syne1	spectrin repeat containing nuclear envelope protein 1	-1.07	7.3E-05
		G00000017993	Abcb10	ATP binding cassette subfamily B member 10	-1.05	8.6E-05
		G00000007545	Angptl4	angiopoietin-like 4	-1.04	4.1E-07
		G00000007990	Adipor2	adiponectin receptor 2	-1.04	1.1E-04
		G00000020134	Upf1	UPF1	-1.03	5.1E-04
		G00000027434	Fitm2	fat storage-inducing transmembrane protein 2	-1.02	1.7E-05
		G00000048315	Eif2ak2	eukaryotic translation initiation factor 2-alpha kinase 2	-1.02	6.7E-05
		G00000005642	Frs2	fibroblast growth factor receptor substrate 2	-1.02	7.7E-04
		G00000014604	Sigmar1	sigma non-opioid intracellular receptor 1	-1.01	4.9E-09
		G00000002175	Clock	clock circadian regulator	-1.01	2.4E-04
		G00000042785	Sesn2	sestrin 2	-1.00	1.7E-05
		G00000023463	Parp9	poly (ADP-ribose) polymerase family	-0.99	3.5E-04
		G00000043377	Fdps	farnesyl diphosphate synthase	-0.99	1.6E-03
		G00000000593	Rev3l	REV3 like	-0.98	2.0E-03
		G00000019283	P2ry2	purinergic receptor P2Y2	-0.98	1.0E-03
		G00000024061	Rarb	retinoic acid receptor	-0.98	2.7E-03
		G00000017220	Tcirg1	T-cell immune regulator 1	-0.98	3.3E-04
		G00000021032	Sphk2	sphingosine kinase 2	-0.97	2.3E-05
		G00000001585	Nrip1	nuclear receptor interacting protein 1	-0.97	1.1E-03
		G00000003882	Cep350	centrosomal protein 350	-0.96	5.2E-04
		G00000005292	Trip11	thyroid hormone receptor interactor 11	-0.96	2.4E-06
		G00000046889	Dbi	diazepam binding inhibitor	-0.96	2.3E-05
		G00000000664	Tpst2	tyrosylprotein sulfotransferase 2	-0.95	2.1E-03
		G00000014900	Crem	cAMP responsive element modulator	-0.95	2.0E-03
		G00000024115	C6	complement C6	-0.93	2.9E-04
		G00000030225	Clpx	caseinolytic mitochondrial matrix peptidase chaperone subunit X	-0.92	3.8E-05
		G00000038012	Commd6	COMM domain containing 6	-0.91	1.8E-04
		G00000007302	Fbn1	fibrillin 1	-0.91	1.4E-03
		G00000018420	Slc22a7	solute carrier family 22 member 7	-0.90	4.1E-04
		G00000002635	Dexi	Dexi homolog	-0.89	1.6E-06
		G00000007728	Gsdmd	gasdermin D	-0.89	2.3E-03
		G00000026942	RGD1311595	similar to KIAA2026 protein	-0.88	2.9E-05
		G00000034066	Hspa8	heat shock protein family A (Hsp70) member 8	-0.87	3.0E-04
		G00000019372	Pc	pyruvate carboxylase	-0.87	8.5E-06
		G00000000177	Plpp2	phospholipid phosphatase 2	-0.87	9.6E-04
		G00000056703	Atrx	ATRX	-0.86	2.0E-04
		G00000016219	Vnn1	vanin 1	-0.86	1.5E-04
		G00000014338	Slc25a25	solute carrier family 25 member 25	-0.86	6.6E-04
		G00000013391	Sorbs2	sorbin and SH3 domain containing 2	-0.84	9.9E-06
		G00000016692	Hsdl2	hydroxysteroid dehydrogenase like 2	-0.83	1.3E-04
		G00000024145	Trim65	tripartite motif-containing 65	-0.83	5.9E-05
		G00000010947	Mmp14	matrix metallopeptidase 14	-0.83	1.7E-03
		G00000018584	Ptma	prothymosin alpha	-0.83	1.5E-05
		G00000008274	Xpc	XPC complex subunit	-0.83	3.6E-04
		G00000011261	Ttc14	tetratricopeptide repeat domain 14	-0.83	2.7E-03
		G00000047386	Smg1	SMG1	-0.82	2.7E-03
		G00000007400	Srebf2	sterol regulatory element binding transcription factor 2	-0.82	4.0E-04
		G00000028801	Gsap	gamma-secretase activating protein	-0.82	2.1E-03
		G00000007700	Inhbc	inhibin subunit beta C	-0.81	5.3E-04
		G00000013178	Cmip	c-Maf-inducing protein	-0.81	6.6E-04
		G00000032394	Tymp	thymidine phosphorylase	-0.80	5.0E-04
		G00000031709	Ppfibp1	PPFIA binding protein 1	-0.79	6.6E-04
		G00000003020	Slc25a47	solute carrier family 25	-0.79	1.5E-03
		G00000019450	Etf1	eukaryotic translation termination factor 1	-0.78	1.6E-03
		G00000010497	RGD1305807	hypothetical LOC298077	-0.77	1.7E-05
		G00000000184	Tmprss6	transmembrane serine protease 6	-0.75	3.7E-04
		G00000004709	Foxn3	forkhead box N3	-0.73	2.2E-04
		G00000007681	Brd3	bromodomain containing 3	-0.72	2.1E-03
		G00000033593	Osbpl9	oxysterol binding protein-like 9	-0.72	7.9E-04
		G00000002212	Hsd17b13	hydroxysteroid (17-beta) dehydrogenase 13	-0.70	5.2E-06
		G00000053550	Itga1	integrin subunit alpha 1	-0.68	1.6E-03
		G00000030700	COX3	cytochrome c oxidase subunit 3	-0.67	2.8E-04
		G00000020425	Stim1	stromal interaction molecule 1	-0.66	1.0E-03
		G00000057814	Nsdhl	NAD(P) dependent steroid dehydrogenase-like	-0.66	2.0E-05
		G00000056371	Pik3ca	phosphatidylinositol-4	-0.66	1.5E-03
		G00000016266	Mphosph10	M-phase phosphoprotein 10	-0.65	2.2E-03
		G00000015441	Il4r	interleukin 4 receptor	-0.65	1.8E-03
		G00000009102	Fermt2	fermitin family member 2	-0.62	2.2E-03
		G00000005015	Rabep1	rabaptin	-0.62	1.8E-03
		G00000020151	Cdh1	cadherin 1	-0.60	2.4E-03
		G00000013135	Ptpn12	protein tyrosine phosphatase	-0.58	2.7E-04
		G00000057623	Copb1	COPI coat complex subunit beta 1	-0.53	4.7E-04
		G00000011140	Prxl2a	peroxiredoxin like 2A	-0.51	2.0E-03
		G00000018849	Tcerg1	transcription elongation regulator 1	-0.51	2.0E-03
		G00000008305	Sc5d	sterol-C5-desaturase	-0.47	2.0E-03
		G00000009263	Ifi27	interferon	-0.47	2.2E-03
		G00000004345	Daam1	dishevelled associated activator of morphogenesis 1	-0.46	2.7E-03
		G00000016963	Trip12	thyroid hormone receptor interactor 12	-0.43	1.7E-03
**ZDF**	**Liver Upregulated**	**Ensembl_ID (ENSRNO)**	**Gene Symbol**	**Gene Name**	**L2FC**	**P-value**
		G00000003119	Gc	GC	0.45	1.1E-03
		G00000000610	Cisd1	CDGSH iron sulfur domain 1	0.46	7.7E-04
		G00000019629	Lamp1	lysosomal-associated membrane protein 1	0.50	8.8E-05
		G00000000701	Iscu	iron-sulfur cluster assembly enzyme	0.51	4.0E-05
		G00000037850	Mtarc2	mitochondrial amidoxime reducing component 2	0.51	6.0E-04
		G00000019048	Sod2	superoxide dismutase 2	0.54	2.8E-04
		G00000007967	Sdhb	succinate dehydrogenase complex iron sulfur subunit B	0.54	1.8E-03
		G00000013928	Dsp	desmoplakin	0.54	1.5E-03
		G00000016794	Phyhd1	phytanoyl-CoA dioxygenase domain containing 1	0.55	2.0E-03
		G00000019626	Slc27a5	solute carrier family 27 member 5	0.55	8.8E-05
		G00000028368	Etnk2	ethanolamine kinase 2	0.55	1.4E-03
		G00000011535	Gcsh	glycine cleavage system protein H	0.56	9.9E-04
		G00000008921	Dynll2	dynein light chain LC8-type 2	0.56	1.5E-03
		G00000030449	Gsta4	glutathione S-transferase alpha 4	0.56	1.1E-03
		G00000018604	Tufm	Tu translation elongation factor	0.59	2.2E-03
		G00000017672	Akr1c14	aldo-keto reductase family 1	0.59	3.1E-04
		G00000020994	Slc25a39	solute carrier family 25	0.59	7.3E-04
		G00000047708	Gstz1	glutathione S-transferase zeta 1	0.59	1.1E-04
		G00000013704	Cps1	carbamoyl-phosphate synthase 1	0.60	5.4E-04
		G00000043404	Uroc1	urocanate hydratase 1	0.60	1.6E-05
		G00000007395	Baat	bile acid CoA:amino acid N-acyltransferase	0.60	5.3E-04
		G00000017577	Bphl	biphenyl hydrolase like	0.60	6.8E-04
		G00000007069	Adhfe1	alcohol dehydrogenase	0.62	4.9E-04
		G00000023538	Aldh5a1	aldehyde dehydrogenase 5 family	0.62	4.9E-04
		G00000006653	Slc38a4	solute carrier family 38	0.62	1.2E-04
		G00000001333	Azgp1	alpha-2-glycoprotein 1	0.62	8.6E-06
		G00000016339	Uox	urate oxidase	0.63	2.8E-05
		G00000061876	Tas1r2	taste 1 receptor member 2	0.63	2.4E-04
		G00000006916	Sardh	sarcosine dehydrogenase	0.63	8.6E-05
		G00000029549	Eci3	enoyl-Coenzyme A delta isomerase 3	0.63	8.9E-04
		G00000048723	Pros1	protein S	0.64	4.9E-04
		G00000009005	Slco2a1	solute carrier organic anion transporter family	0.64	2.7E-05
		G00000007839	Slc16a7	solute carrier family 16 member 7	0.64	8.2E-04
		G00000010389	Ndrg2	NDRG family member 2	0.65	5.7E-04
		G00000014165	Ssr1	signal sequence receptor subunit 1	0.65	1.0E-04
		G00000029735	Pid1	phosphotyrosine interaction domain containing 1	0.65	1.9E-03
		G00000033466	Apon	apolipoprotein N	0.65	1.1E-03
		G00000000158	Cdo1	cysteine dioxygenase type 1	0.65	6.6E-06
		G00000008364	Cat	catalase	0.67	1.1E-03
		G00000061883	Aqp9	aquaporin 9	0.68	1.3E-03
		G00000021916	Slc16a12	solute carrier family 16	0.68	2.5E-03
		G00000007743	Mgst1	microsomal glutathione S-transferase 1	0.68	1.8E-05
		G00000003653	Fh	fumarate hydratase	0.68	1.6E-03
		G00000013223	Fah	fumarylacetoacetate hydrolase	0.69	2.4E-04
		G00000014700	Ttc36	tetratricopeptide repeat domain 36	0.69	8.4E-05
		G00000030862	Atp6v1h	ATPase H+ transporting V1 subunit H	0.69	4.9E-04
		G00000030667	Ppm1b	protein phosphatase	0.71	4.7E-06
		G00000004139	Ndel1	nudE neurodevelopment protein 1-like 1	0.72	3.8E-05
		G00000007927	Mettl7b	methyltransferase like 7B	0.72	5.0E-05
		G00000004147	Abca8a	ATP-binding cassette	0.73	1.4E-03
		G00000029726	Gstm1	glutathione S-transferase mu 1	0.74	1.3E-03
		G00000003370	Otc	ornithine carbamoyltransferase	0.74	6.8E-06
		G00000013039	Add1	adducin 1	0.74	4.2E-04
		G00000014727	Fahd1	fumarylacetoacetate hydrolase domain containing 1	0.75	4.2E-04
		G00000059463	Slc39a1	solute carrier family 39 member 1	0.76	1.6E-03
		G00000004302	Pah	phenylalanine hydroxylase	0.76	3.4E-07
		G00000029651	Rdh16	retinol dehydrogenase 16	0.76	8.2E-04
		G00000028746	Gsto1	glutathione S-transferase omega 1	0.77	3.2E-04
		G00000018426	NEWGENE_2134	apolipoprotein C1	0.77	1.3E-06
		G00000001053	Tmed2	transmembrane p24 trafficking protein 2	0.77	6.7E-04
		G00000016173	Cyp1a2	cytochrome P450	0.77	6.7E-04
		G00000004089	Enpp2	ectonucleotide pyrophosphatase/phosphodiesterase 2	0.78	3.5E-04
		G00000042274	Fbxo31	F-box protein 31	0.78	2.3E-03
		G00000000186	Tst	thiosulfate sulfurtransferase	0.78	8.6E-05
		G00000048812	Gpx1	glutathione peroxidase 1	0.79	5.0E-04
		G00000047986	Sult2a1	sulfotransferase family 2A member 1	0.79	2.5E-03
		G00000006345	Sec61b	SEC61 translocon subunit beta	0.79	6.2E-04
		G00000009779	Krt8	keratin 8	0.79	2.2E-03
		G00000006623	Cd302	CD302 molecule	0.80	1.5E-04
		G00000005987	Suox	sulfite oxidase	0.81	1.1E-03
		G00000061890	Ust5r	integral membrane transport protein UST5r	0.81	2.3E-04
		G00000020879	Nags	N-acetylglutamate synthase	0.81	3.3E-04
		G00000008902	Pon1	paraoxonase 1	0.82	9.7E-07
		G00000018904	Dtymk	deoxythymidylate kinase	0.82	2.1E-03
		G00000023116	Agmo	alkylglycerol monooxygenase	0.82	4.0E-05
		G00000047816	Ccs	copper chaperone for superoxide dismutase	0.84	1.3E-04
		G00000012142	Glyat	glycine-N-acyltransferase	0.84	5.6E-07
		G00000021206	Plaat3	phospholipase A and acyltransferase 3	0.84	7.5E-04
		G00000012962	Nudt16	nudix hydrolase 16	0.85	1.9E-04
		G00000050315	Dcxr	dicarbonyl and L-xylulose reductase	0.86	2.9E-06
		G00000000024	Hebp1	heme binding protein 1	0.86	2.7E-04
		G00000000386	Pbld1	phenazine biosynthesis-like protein domain containing 1	0.87	1.3E-05
		G00000007378	Acox2	acyl-CoA oxidase 2	0.87	7.0E-05
		G00000003307	Gcdh	glutaryl-CoA dehydrogenase	0.87	2.2E-08
		G00000002205	Ociad1	OCIA domain containing 1	0.87	1.4E-03
		G00000014645	Aldh7a1	aldehyde dehydrogenase 7 family	0.88	8.2E-08
		G00000008638	Angptl3	angiopoietin-like 3	0.88	2.9E-09
		G00000011351	Mat1a	methionine adenosyltransferase 1A	0.89	3.6E-05
		G00000009421	Ivd	isovaleryl-CoA dehydrogenase	0.89	1.9E-09
		G00000036894	Cisd3	CDGSH iron sulfur domain 3	0.89	4.0E-04
		G00000014128	Ecsit	ECSIT signaling integrator	0.90	1.6E-03
		G00000017619	Aldh1a1	aldehyde dehydrogenase 1 family	0.90	3.1E-05
		G00000018662	Amacr	alpha-methylacyl-CoA racemase	0.90	3.9E-07
		G00000020000	Tmem219	transmembrane protein 219	0.90	5.2E-04
		G00000001957	Sult1e1	sulfotransferase family 1E member 1	0.90	2.8E-06
		G00000051860	Rnase4	ribonuclease A family member 4	0.91	1.3E-09
		G00000014160	Tcp1	t-complex 1	0.91	2.2E-04
		G00000048114	Echdc3	enoyl CoA hydratase domain containing 3	0.91	2.7E-07
		G00000003291	Creg1	cellular repressor of E1A-stimulated genes 1	0.92	1.3E-07
		G00000008837	Ass1	argininosuccinate synthase 1	0.92	7.7E-04
		G00000018159	Anxa4	annexin A4	0.92	2.3E-04
		G00000010993	Dpm1	dolichyl-phosphate mannosyltransferase subunit 1	0.92	9.1E-04
		G00000019982	Ethe1	ETHE1	0.92	2.4E-05
		G00000023177	Esrp2	epithelial splicing regulatory protein 2	0.93	9.8E-07
		G00000013409	Gclm	glutamate cysteine ligase	0.93	3.0E-04
		G00000001806	Fetub	fetuin B	0.93	2.9E-04
		G00000017291	Sord	sorbitol dehydrogenase	0.94	7.2E-09
		G00000053362	Gabarapl1	GABA type A receptor associated protein like 1	0.94	1.4E-07
		G00000021174	Macrod1	mono-ADP ribosylhydrolase 1	0.95	7.1E-05
		G00000014268	Abca2	ATP binding cassette subfamily A member 2	0.95	9.8E-04
		G00000049771	Gstt1	glutathione S-transferase theta 1	0.96	8.4E-05
		G00000011226	Timm8a1	translocase of inner mitochondrial membrane 8A1	0.96	4.5E-06
		G00000005175	Sgpp1	sphingosine-1-phosphate phosphatase 1	0.97	2.0E-03
		G00000049464	Cyp2c13	cytochrome P450	0.97	6.0E-10
		G00000002210	Hsd17b11	hydroxysteroid (17-beta) dehydrogenase 11	0.97	4.4E-10
		G00000012786	Pgrmc1	progesterone receptor membrane component 1	0.99	1.2E-07
		G00000004327	Ddc	dopa decarboxylase	0.99	4.8E-05
		G00000046357	Adh5	alcohol dehydrogenase 5 (class III)	0.99	1.2E-11
		G00000054049	Prelid2	PRELI domain containing 2	0.99	7.6E-04
		G00000004442	Dglucy	D-glutamate cyclase	0.99	1.6E-03
		G00000014876	Lpin2	lipin 2	1.00	3.9E-04
		G00000012911	Erlin1	ER lipid raft associated 1	1.00	6.8E-04
		G00000055314	Msrb1	methionine sulfoxide reductase B1	1.00	1.1E-07
		G00000006619	Dnajc9	DnaJ heat shock protein family (Hsp40) member C9	1.01	6.5E-04
		G00000018937	Gstm7	glutathione S-transferase	1.01	1.6E-04
		G00000027016	Cobll1	cordon-bleu WH2 repeat protein-like 1	1.01	1.4E-04
		G00000046007	Cldn3	claudin 3	1.02	2.8E-04
		G00000033609	Irx1	iroquois homeobox 1	1.02	2.0E-03
		G00000017777	Ahcy	adenosylhomocysteinase	1.02	1.5E-05
		G00000019180	Acsl4	acyl-CoA synthetase long-chain family member 4	1.02	1.0E-08
		G00000022932	Serhl2	serine hydrolase-like 2	1.03	1.5E-04
		G00000016484	Gstk1	glutathione S-transferase kappa 1	1.03	1.5E-07
		G00000003620	Fmo3	flavin containing dimethylaniline monoxygenase 3	1.04	1.7E-05
		G00000032895	Cyp4f4	cytochrome P450	1.04	5.0E-08
		G00000032737	F7	coagulation factor VII	1.05	2.1E-04
		G00000023816	Aph1a	aph-1 homolog A	1.05	1.6E-03
		G00000015205	Cyb5a	cytochrome b5 type A	1.06	9.6E-07
		G00000008079	Ugp2	UDP-glucose pyrophosphorylase 2	1.06	4.1E-08
		G00000011559	Cnn3	calponin 3	1.07	5.6E-05
		G00000013484	Gsta1	glutathione S-transferase alpha-1	1.07	4.2E-10
		G00000050595	Mup5	major urinary protein 5	1.07	1.5E-04
		G00000026775	Pmpca	peptidase	1.08	3.9E-04
		G00000001338	Hpd	4-hydroxyphenylpyruvate dioxygenase	1.08	7.7E-06
		G00000001618	Ripk4	receptor-interacting serine-threonine kinase 4	1.09	2.3E-03
		G00000000768	Ubd	ubiquitin D	1.10	2.4E-05
		G00000007508	Lrtm2	leucine-rich repeats and transmembrane domains 2	1.10	1.8E-08
		G00000031769	Chchd7	coiled-coil-helix-coiled-coil-helix domain containing 7	1.10	4.4E-04
		G00000013291	Cyp2c23	cytochrome P450	1.10	4.4E-07
		G00000017188	Cyp27a1	cytochrome P450	1.11	1.7E-08
		G00000058327			1.13	7.4E-04
		G00000025079	Fam126b	family with sequence similarity 126	1.13	4.7E-04
		G00000061215	Crym	crystallin	1.14	2.1E-04
		G00000017752	Mccc2	methylcrotonoyl-CoA carboxylase 2	1.16	1.8E-05
		G00000016166	Pdlim1	PDZ and LIM domain 1	1.16	7.7E-07
		G00000010079	Ca3	carbonic anhydrase 3	1.17	4.7E-10
		G00000013728	Polg2	DNA polymerase gamma 2	1.17	6.2E-04
		G00000062298	Rpl13a	ribosomal protein L13A	1.19	2.6E-03
		G00000013751	Plpbp	pyridoxal phosphate binding protein	1.19	2.3E-06
		G00000001442	Por	cytochrome p450 oxidoreductase	1.19	1.2E-09
		G00000042253	Ecd	ecdysoneless cell cycle regulator	1.20	6.0E-04
		G00000020254	Per2	period circadian regulator 2	1.20	3.1E-04
		G00000007949	Rgn	regucalcin	1.21	8.8E-08
		G00000003253	Qdpr	quinoid dihydropteridine reductase	1.21	3.4E-09
		G00000003515	Ephx1	epoxide hydrolase 1	1.22	7.9E-07
		G00000011039	Gch1	GTP cyclohydrolase 1	1.23	2.8E-07
		G00000038746	Bco2	beta-carotene oxygenase 2	1.24	6.3E-07
		G00000005861	Hsd11b1	hydroxysteroid 11-beta dehydrogenase 1	1.24	4.3E-09
		G00000000588	Slc16a10	solute carrier family 16 member 10	1.24	1.6E-05
		G00000020202	Asrgl1	asparaginase and isoaspartyl peptidase 1	1.25	2.7E-03
		G00000017826	Mtrr	5-methyltetrahydrofolate-homocysteine methyltransferase reductase	1.26	1.6E-03
		G00000033700	Bud23	BUD23	1.27	1.7E-03
		G00000000281	Prodh1	proline dehydrogenase 1	1.28	2.1E-11
		G00000042084	Acsm2	acyl-CoA synthetase medium-chain family member 2	1.30	3.4E-09
		G00000006972	Zfp189	zinc finger protein 189	1.30	1.5E-03
		G00000027784	Tsku	tsukushi	1.32	7.2E-04
		G00000012387	Glyatl2	glycine-N-acyltransferase-like 2	1.33	7.3E-07
		G00000011714	Sat2	spermidine/spermine N1-acetyltransferase family member 2	1.33	6.9E-05
		G00000045799	Rup2	urinary protein 2	1.34	7.8E-04
		G00000021924	Cyp2c22	cytochrome P450	1.35	9.3E-08
		G00000018494	Ppp1r3c	protein phosphatase 1	1.36	4.1E-11
		G00000004693	Pbx1	PBX homeobox 1	1.36	1.4E-03
		G00000001258	Snx8	sorting nexin 8	1.37	2.0E-04
		G00000020698	Rnd2	Rho family GTPase 2	1.37	6.0E-05
		G00000051227			1.38	6.2E-04
		G00000052810	Cyp2c11	cytochrome P450	1.39	2.0E-11
		G00000012436	Adh6	alcohol dehydrogenase 6 (class V)	1.41	4.4E-15
		G00000015936	Gng5	G protein subunit gamma 5	1.41	2.6E-03
		G00000018413	Per3	period circadian regulator 3	1.42	1.6E-04
		G00000016967	Hfe	homeostatic iron regulator	1.42	2.9E-07
		G00000001376	Mettl7a	methyltransferase like 7A	1.43	3.1E-04
		G00000056940	Cited2	Cbp/p300-interacting transactivator	1.44	1.5E-11
		G00000015002	Abhd15	abhydrolase domain containing 15	1.44	1.5E-04
		G00000032959	Adh7	alcohol dehydrogenase 7 (class IV)	1.45	7.6E-09
		G00000050232	LOC680406	similar to Urinary protein 2 precursor (RUP-2)	1.46	1.5E-09
		G00000020700	Rnaseh2c	ribonuclease H2	1.48	7.7E-04
		G00000011635	Ces2e	carboxylesterase 2E	1.49	2.9E-08
		G00000015354	Aox1	aldehyde oxidase 1	1.54	2.6E-12
		G00000061450	Homer2	homer scaffold protein 2	1.54	2.5E-05
		G00000009629	Car2	carbonic anhydrase 2	1.55	2.9E-05
		G00000042111	Sult1c2a	sulfotransferase family	1.55	2.3E-03
		G00000057072	Slc12a3	solute carrier family 12 member 3	1.55	3.1E-04
		G00000004009	Xpnpep2	X-prolyl aminopeptidase 2	1.57	1.1E-08
		G00000013313	Nceh1	neutral cholesterol ester hydrolase 1	1.57	8.8E-07
		G00000015438	LOC501233	LRRGT00080	1.58	1.2E-13
		G00000015076	Cyp26b1	cytochrome P450	1.62	6.3E-04
		G00000016456	Il33	interleukin 33	1.65	4.2E-18
		G00000001766	Tfrc	transferrin receptor	1.67	1.1E-04
		G00000011718	C1rl	complement C1r subcomponent like	1.68	1.2E-06
		G00000013949	Idh2	isocitrate dehydrogenase (NADP(+)) 2	1.68	2.3E-16
		G00000018740	Ugt1a6	UDP glucuronosyltransferase family 1 member A6	1.69	6.1E-14
		G00000016807	Oat	ornithine aminotransferase	1.72	1.2E-05
		G00000025418	Armc9	armadillo repeat containing 9	1.74	4.5E-04
		G00000023778	Gcnt2	glucosaminyl (N-acetyl) transferase 2 (I blood group)	1.77	6.8E-05
		G00000056596	Alas1	5'-aminolevulinate synthase 1	1.80	2.3E-15
		G00000046643	Cyp3a9	cytochrome P450	1.82	5.3E-04
		G00000003260	Nr1i3	nuclear receptor subfamily 1	1.84	6.4E-05
		G00000001158	Abcg1	ATP binding cassette subfamily G member 1	1.86	3.0E-05
		G00000020250	Pcgf6	polycomb group ring finger 6	1.88	9.2E-04
		G00000006420	Rbm38	RNA binding motif protein 38	1.89	2.0E-04
		G00000012458	Cyp2e1	cytochrome P450	1.91	1.3E-19
		G00000002258	Tmem150c	transmembrane protein 150C	1.94	9.3E-05
		G00000013982	Hsd17b2	hydroxysteroid (17-beta) dehydrogenase 2	1.94	5.1E-04
		G00000021027	Dbp	D-box binding PAR bZIP transcription factor	1.94	3.2E-05
		G00000004437	Map2k6	mitogen-activated protein kinase kinase 6	2.07	1.1E-08
		G00000032246	Acsm3	acyl-CoA synthetase medium-chain family member 3	2.19	2.3E-16
		G00000014490	Bdh2	3-hydroxybutyrate dehydrogenase 2	2.21	2.5E-14
		G00000036687	Alyref	Aly/REF export factor	2.23	8.9E-04
		G00000015519	Ces1d	carboxylesterase 1D	2.24	6.9E-32
		G00000009598	Ncaph2	non-SMC condensin II complex	2.41	3.8E-05
		G00000043131	LOC100360095	urinary protein 1-like	2.43	4.9E-19
		G00000034191	Fmo1	flavin containing dimethylaniline monoxygenase 1	2.46	2.8E-25
		G00000005985	Kcnma1	potassium calcium-activated channel subfamily M alpha 1	2.82	1.6E-04
		G00000011250	Inmt	indolethylamine N-methyltransferase	2.90	3.9E-21
		G00000058904	Tex13b	testis expressed 13B	3.10	5.9E-15
		G00000012772	Nqo1	NAD(P)H quinone dehydrogenase 1	3.11	1.5E-12
		G00000001388	Sds	serine dehydratase	3.29	1.7E-09
		G00000056847	Gsta3	glutathione S-transferase alpha 3	3.40	3.1E-21
		G00000001242	Gstt3	glutathione S-transferase	3.66	1.2E-85
		G00000051912	Acnat2	acyl-coenzyme A amino acid N-acyltransferase 2	3.74	8.9E-05
		G00000009488	Cyp7a1	cytochrome P450 family 7 subfamily A member 1	4.19	1.4E-18
**Lean**	**Liver Downregulated**	**Ensembl_ID (ENSRNO)**	**Gene Symbol**	**Gene Name**	**L2FC**	**P-value**
		G00000029668	Wfdc21	WAP four-disulfide core domain 21	-2.72	1.3E-04
		G00000020480	Fads1	fatty acid desaturase 1	-2.53	4.0E-08
		G00000006859	Insig1	insulin induced gene 1	-2.32	3.2E-05
		G00000057557	Prlr	prolactin receptor	-2.27	2.3E-04
		G00000055909	Apoa4	apolipoprotein A4	-1.93	2.3E-04
		G00000030154	Cyp4a2	cytochrome P450	-1.75	2.2E-08
		G00000019776	Sh3gl3	SH3 domain containing GRB2 like 3	-1.62	8.9E-05
		G00000046889	Dbi	diazepam binding inhibitor	-1.61	1.1E-05
		G00000014702	Elovl2	ELOVL fatty acid elongase 2	-1.60	1.8E-05
		G00000032297	Msmo1	methylsterol monooxygenase 1	-1.56	3.6E-05
		G00000007234	Cyp51	cytochrome P450	-1.56	4.9E-05
		G00000020989	Tm7sf2	transmembrane 7 superfamily member 2	-1.45	6.6E-05
**Lean**	**Liver Upregulated**	**Ensembl_ID (ENSRNO)**	**Gene Symbol**	**Gene Name**	**L2FC**	**P-value**
		G00000001376	Mettl7a	methyltransferase like 7A	1.16	1.7E-04
		G00000048114	Echdc3	enoyl CoA hydratase domain containing 3	1.17	1.2E-04
		G00000023116	Agmo	alkylglycerol monooxygenase	1.21	1.7E-04
		G00000002643	Ugdh	UDP-glucose 6-dehydrogenase	1.29	7.6E-05
		G00000015354	Aox1	aldehyde oxidase 1	1.33	1.2E-04
		G00000004089	Enpp2	ectonucleotide pyrophosphatase/phosphodiesterase 2	1.34	9.6E-05
		G00000034191	Fmo1	flavin containing dimethylaniline monoxygenase 1	1.37	1.7E-04
		G00000013291	Cyp2c23	cytochrome P450	1.47	1.1E-04
		G00000003809	Sat1	spermidine/spermine N1-acetyl transferase 1	1.58	3.9E-05
		G00000018740	Ugt1a6	UDP glucuronosyltransferase family 1 member A6	1.80	2.6E-05
		G00000015519	Ces1d	carboxylesterase 1D	1.94	5.6E-10
		G00000033570	Arhgap8	Rho GTPase activating protein 8	2.03	1.5E-04
		G00000051912	Acnat2	acyl-coenzyme A amino acid N-acyltransferase 2	2.05	1.1E-04
		G00000001158	Abcg1	ATP binding cassette subfamily G member 1	2.21	1.1E-04
		G00000001388	Sds	serine dehydratase	2.29	8.0E-06
		G00000047613	AABR07048463.1	AABR07048463.1	2.34	1.4E-06
		G00000013552	Scd	stearoyl-CoA desaturase	2.36	4.8E-06
		G00000001242	Gstt3	glutathione S-transferase	2.93	1.4E-09
		G00000021924	Cyp2c22	cytochrome P450	3.22	5.2E-15
		G00000009488	Cyp7a1	cytochrome P450 family 7 subfamily A member 1	3.36	1.5E-09

^1^All genes were analyzed using DESeq2 for differential analysis.

^2^Abbreviations used: ZDF, Zucker Diabetic Fatty; L2FC, log2 fold change; PFC, prefrontal cortex.

^3^ Benjamini-Hochberg adjusted P-values controlling for false discovery rate at 5%, where P< 0.05 was considered significant.

We previously demonstrated that WE consumption for 8 wk is effective at improving serum vitamin D status and providing nephroprotective benefits [17, 18]; however, despite our gene expression findings in this study we still have yet to elucidate the mechanism underlying how WE consumption leads to decreased weight gain [28, 29]. We also identified that ZDF rats fed WE upregulated 11 genes involved in glutathione metabolism in the liver and kidney. In the PFC, WE consumption had differing effects whereby in the lean PFC, WE consumption did not change the transcriptome, whereas in the ZDF rats WE consumption strongly downregulated the expression of 2, AY172581 exon transcripts. These exon transcripts have yet to be characterized and future proteomic studies may reveal their biological importance. Across both genotypes, the most significantly altered genes were involved in the Kyoto Encyclopedia of Genes and Genomes (KEGG) pathways of: glutathione metabolism, metabolic pathways, steroid biosynthesis, and cholesterol metabolism. After controlling for the genetic background differences of our ZDF rats, a combined analysis indicated that 428 unique genes were differentially expressed across these tissues as a product of WE consumption. Moreover, 13 different glutathione metabolism genes were significantly upregulated across the liver and kidney in both genotypes suggesting that increased whole egg consumption, may increase glutathione metabolism independent of T2DM, and attenuate the decreased glutathione metabolism during diabetes.

**Fig 1:**
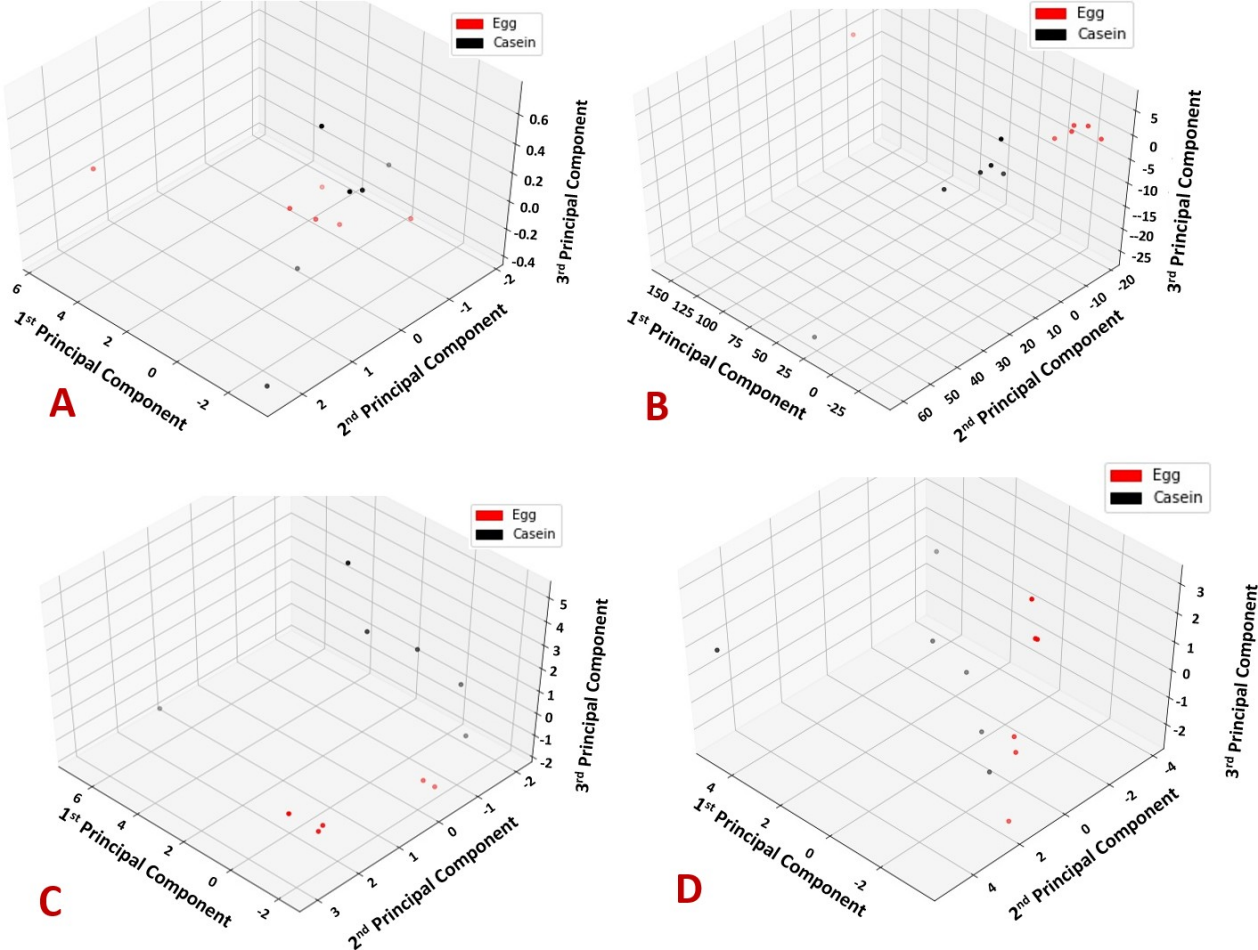
Principle component analysis. Samples in the first three principle component space are colored in red or black for either WE or CAS, respectively. Each panel displays PCA results using microRNA data or mRNA data are colored by dietary treatment groups with A) ZDF microRNA, B) ZDF mRNA, C) lean microRNA, D) lean mRNA.

**Fig 2:**
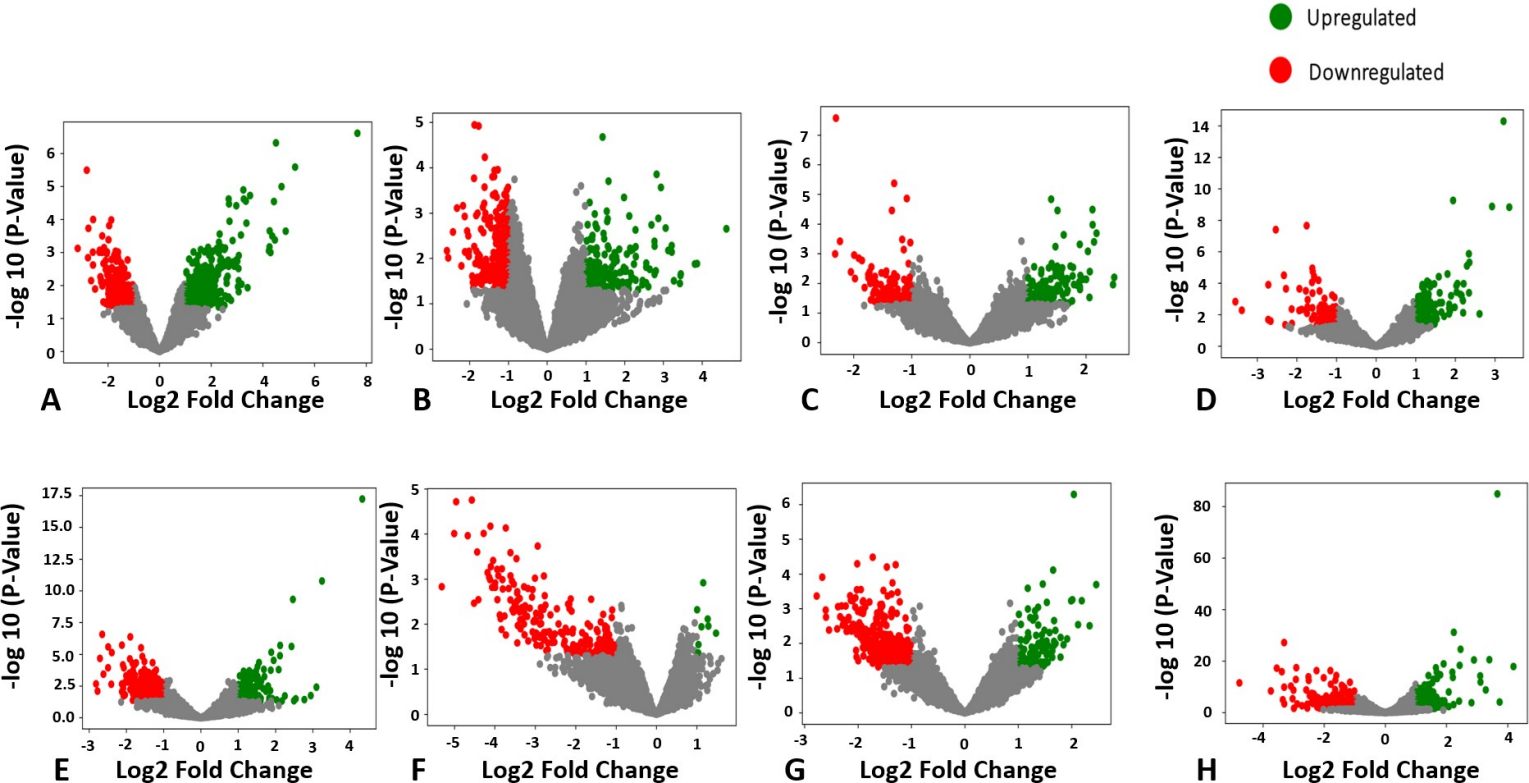
Volcano plots. Genes upregulated (green) or downregulated (red) by WE consumption, correspond to a 1.5 decrease or increase in log fold changes. Each panel corresponds to a tissue in a given genotype: A) lean adipose; B) lean PFC; C) lean kidney; D) lean liver; E) ZDF adipose; F) ZDF PFC; G) ZDF kidney; and H) ZDF liver.

To visualize the global differences in the transcriptomes based on dietary treatment, we performed principal component analysis (PCA) and generated volcano plots for genes that exhibited ≥1.5-fold change, respectively. **Fig 1**displays the samples in a three-dimensional principal component space, whereby samples are colored in red or black to distinguish either WE or CAS, respectively. In the mRNA samples, rats on the same dietary treatment (i.e. black or red) clustered together, while animals belonging to different dietary treatments separated, indicating distinctly different patterns across global mRNA expression. These results were further visualized using volcano plots for each tissue as presented in **Fig 2.** These volcano plots demonstrate the degree to which genes were upregulated or downregulated across each tissue. For instance, volcano plots indicate a relatively equal number of upregulated and downregulated genes in the lean PFC following WE consumption, whereas WE consumption primarily resulted in downregulated gene expression in the ZDF PFC.

During T2DM, reports indicate that genes within the oxidative stress-related pathways upregulated [26]. Evans et al. suggested that oxidative stress was driven by the hyperglycemic environment concomitant with increased concentrations of free fatty acids in the plasma [26]. Corbett et al. [30] reported that protective antioxidant genes such as glutathione peroxidase are downregulated during T2DM, and both glutathione s-transferases (GSTs) and glutathione-dependent enzymes are important in the regulation of pathophysiological alterations in numerous chronic diseases, especially T2DM [30]. Previous work has shown that dietary intervention with direct glutathione supplementation was protective against diabetic nephropathy in an insulin dependent streptozotocin-induced T1DM model [27]. This current study provides new transcriptomic evidence supporting our previous report demonstrating that WE consumption protects against diabetic nephropathy, where WE consumption leads to altered gene expression in the kidney. In this study, we noted that the strongest alterations in glutathione metabolism were in the liver, potentially because hepatic glutathione is produced at much higher concentrations (10 mM), whereas intracellular glutathione concentrations are approximately 1–2 mM [31]. This body of previous work is important in relation to our findings that several GSTs and glutathione-dependent enzymes are significantly altered during WE consumption in lean controls and during diabetes in the kidneys and livers across both genotypes. Future mechanistic studies identifying the beneficial impact of these two enzymes in chronic diseases like T2DM are warranted.

Outside of the glutathione pathways, we also observed that there were significant differences in early growth response-1 (*Egr-1*) gene expression following WE consumption. Egr-1 has been implicated in the onset of insulin-resistance, as previous studies in insulin-resistant T2DM mice identified that loss of function in Egr-1 restores insulin sensitivity via increased phosphorylation of the insulin receptor substrate-1 tyrosine kinase [32]. Notably, we observed a 30% decrease in hepatic *Egr-1* expression in the ZDF rats fed WE. This is an interesting finding as research by Garnett et al. [33] determined that exposing beta cells to hyperglycemic conditions resulted in a temporal and dose-dependent increase in *Egr-1* transcription and translation. Furthermore, Egr-1 null mice are known for their inability of displaying diabetic and obese phenotypes [34] owing to their increased energy expenditure. These data suggest that consumption of WE may lead to altered Egr-1 expression which may play a key role in regulating energy expenditure.

We also demonstrated that WE consumption resulted in tissue-specific alterations in gene expression and that there were distinct transcriptomic differences between genotypes. WE consumption did not influence gene expression in the PFC of lean animals, while 2 genes were significantly altered in the ZDF PFC. There were more stark differences when comparing the liver tissues between the two genotypes, where more than 400 genes were altered in ZDF livers that were not altered in the liver of lean controls. It has been shown that T2DM impacts a variety of tissues [1] but previous studies have provided very little evidence of how T2DM alters the nutrigenomic responses to foods in specific tissues. It is still unknown which specific egg components lead to phenotypic differences in gene expression and future studies should focus on identifying the specific egg constituents that mediate these gene expression differences. These collective findings are likely mediated through the alteration of several genes; therefore, we aimed to further examine microRNA changes involved in the underlying progression of T2DM during WE consumption.

### MicroRNA sequencing differential expression

We examined if endogenously expressed microRNA profiles in the adipose, liver, kidney, and prefrontal cortex tissues would be altered following 8 wk consumption of dietary WE. Differential expression analyses of the ZDF microRNA data resulted in 1 differentially expressed microRNA in the adipose tissue, none in the liver, none in the kidney and 2 in the PFC that surpassed multiple testing correction. Among the lean rats, there were 2 marginally differentially expressed microRNAs in the adipose tissue, 4 in the liver, none in the kidney and none in the PFC that survived multiple testing correction. **Table 2**presents the differentially expressed microRNAs in the adipose, liver, kidney, and PFC tissues across both genotypes. **S3 Table** contains results from DESeq2 with the results for each microRNA across all four tissues and raw microRNA read counts are contained in **S4 Table**.

**Table 2:** Differentially expressed microRNAs in WE vs CAS-based diets in ZDF rats and their lean controls^1^^-^^3^.

Genotype	Tissue	MicroRNA	L2FC	Non adjusted p-value	*P*-value^3^
ZDF	Adipose Downregulated	rno-miR-221-3p	-1.60	9.53E-05	0.007
ZDF	PFC Upregulated	rno-miR-29a-3p	0.59	0.0001	0.022
ZDF	PFC Upregulated	rno-miR-151-5p	0.89	0.0005	0.036
Lean	Adipose Downregulated	rno-miR-125a-5p	-1.48	0.0022	0.069
Lean	Adipose Downregulated	rno-miR-125b-5p	-1.78	0.0029	0.069
Lean	Liver Upregulated	rno-miR-9a-5p	1.89	9.08E-05	0.0063
Lean	Liver Upregulated	rno-miR-181a-5p	1.10	0.0007	0.024
Lean	Liver Upregulated	rno-miR-10b-5p	1.37	0.0011	0.024
Lean	Liver Downregulated	rno-miR-192-5p	-0.57	0.0013	0.024

^1^All miRNAs were analyzed using DESeq2 for differential analysis.

^2^Abbreviations used: ZDF, Zucker Diabetic Fatty; WE, whole egg; CAS, casein; L2FC, log2 fold change; and PFC, prefrontal cortex.

^3^Benjamini-Hochberg adjusted P-values controlling for false discovery rate at 5%, where P< 0.05 was considered significant.

Based on the microRNA sequencing analysis, 9 microRNAs were differentially expressed following multiple testing correction. Several of these microRNAs have been previously correlated with gestational diabetes or show to be altered in the plasma of individuals with diabetes. Very few studies to date have examined the tissue-specific changes of endogenous microRNA expression in response to dietary patterns and this is the first study to demonstrate that endogenous microRNA expression in the liver, adipose, and PFC can be altered following 8 wks of WE consumption. Future studies should focus on identifying if similar foods such as quail eggs alter microRNA expression in these tissues and determine the smallest effective dosage of egg required to recapitulate these changes in microRNAs.

### Mapping between microRNAs and target genes

Next, we sought to determine if these significantly altered microRNAs were responsible for the tissue-specific differential expression of their predicted target genes. MicroRNA mapping analyses of the differentially expressed microRNAs and their target genes demonstrates that in each of the tissues with differentially expressed microRNAs, key target genes of these microRNAs were altered. For instance, in the lean liver microRNA-181a-3p was upregulated and two of its mRNA target genes were differentially expressed, Cytochrome P450 Family 7 Subfamily A Member 1 (*Cyp7a1*) and stearoyl-CoA desaturase (*Scd*). Similarly, in the lean adipose, microRNA-125b-5p was downregulated while its target gene phosphoglycolate phosphatase (*Pgp)* was upregulated. The microRNAs in the PFC and kidney tissue did not map to any differentially expressed genes. **Table 3**summarizes the mapping between microRNAs and their gene targets.

**Table 3:** Differentially expressed microRNAs and their corresponding target genes that were differentially regulated by dietary WE consumption^1^^-^^3^.

MicroRNA	Tissue	Gene Name	L2FC	Non-adj *p*-value	Human Symbol	Ensembl Rat ID (RNOG)	Rat Symbol	Gene Name
miR- 125b-5p (down regulated)	lean adipose	Pgp	2.69	0.00003	PGP	00000009536	Pgp	phosphoglycolate phosphatase
rno-miR- 181a-5p (up regulated)	lean liver	CYP7A1	3.36	0.000000001	Cyp7a1	00000009488	Cyp7a1	Cytochrome P450 Family 7 Subfamily A Member 1
rno-miR- 181a-5p (up regulated)	lean liver	Scd	2.35	0.0000048	Scd	00000013552	SCD	stearoyl-CoA desaturase

^1^All miRNAs were analyzed using DESeq2 for differential analysis.

^2^Abbreviations used: miR, microRNAS; rno, rattus norvegicus; WE, whole egg; and L2FC, log2 fold change.

^3^Gene targets for *Rattus Norvegicus* and humans were determined by DRSC Integrative Ortholog Prediction Tool.

While examining the relationship between significantly altered microRNAs and their target genes, we identified that in the livers of lean rats fed WE, the upregulated microRNA-181a-5p affected target genes involved in steroid hormone biosynthesis such as *Cyp7a1* and *Scd*. Notably, only *Cyp7a1* was upregulated in the liver of ZDF rats fed the WE-based diet while both *Scd* and *Cyp7a1* were upregulated in the livers of lean control rats. In rodent models of diabetes, liver expression of *Cyp7a1* has been shown to be decreased and thought to play a key role in regulating whole body energy homeostasis [35]. Similarly, transgenic mice overexpressing *Cyp7a1* were shown to become resistant to weight gain and fatty liver disease [35]. Experiments examining the role of *Scd* in rat hepatocytes has demonstrated that *Scd* expression regulates hepatic insulin resistance during diabetes [36], but very few studies have determined the expression of *Scd* genes in the context of dietary consumption. Based on the data, WE consumption more strongly upregulated hepatic expression of *Cyp7a1* in ZDF animals than in the lean controls and this might suggest that WE consumption can prevent or reverse the loss of hepatic *Cyp7a1* expression due to diabetes.

In our lean rats, we also identified that microRNA-125b-5p was downregulated in adipose tissue where its gene target *Pgp* was strongly upregulated. *Pgp* is known to hydrolyze glycerol-3-phosphate into glycerol, and overexpression experiments in rodents showed that upregulation of Pgp leads to a reduction in body weight gain and improves hepatic glucose regulation [37]. Additionally, we observed the upregulation of liver microRNA-9a-5p, which has been correlated with gestational diabetes in humans [38]. While the gene targets of microRNA-9a-5p were not differentially expressed in the liver, future studies should look into whether endogenous microRNA expression fluctuates in response to consuming other eggs, such as quail eggs, or egg yolk alone.

### KEGG and GO functional enrichment analysis

To further examine the molecular function of the identified DEGs, KEGG pathway analysis indicated that the most prevalent pathways influenced by dietary WE across multiple tissues in the ZDF rats were: glutathione metabolism; oxidation-reduction; metabolism of xenobiotics; steroid hormone biosynthesis; and fatty acid synthesis pathways. In the livers of lean control rats, the most significantly expressed pathways included metabolic pathways and retinol metabolism. All the differentially expressed genes that map to KEGG and gene ontology (GO) pathways analyses are presented in **S5 Table.**

**Fig 3:**
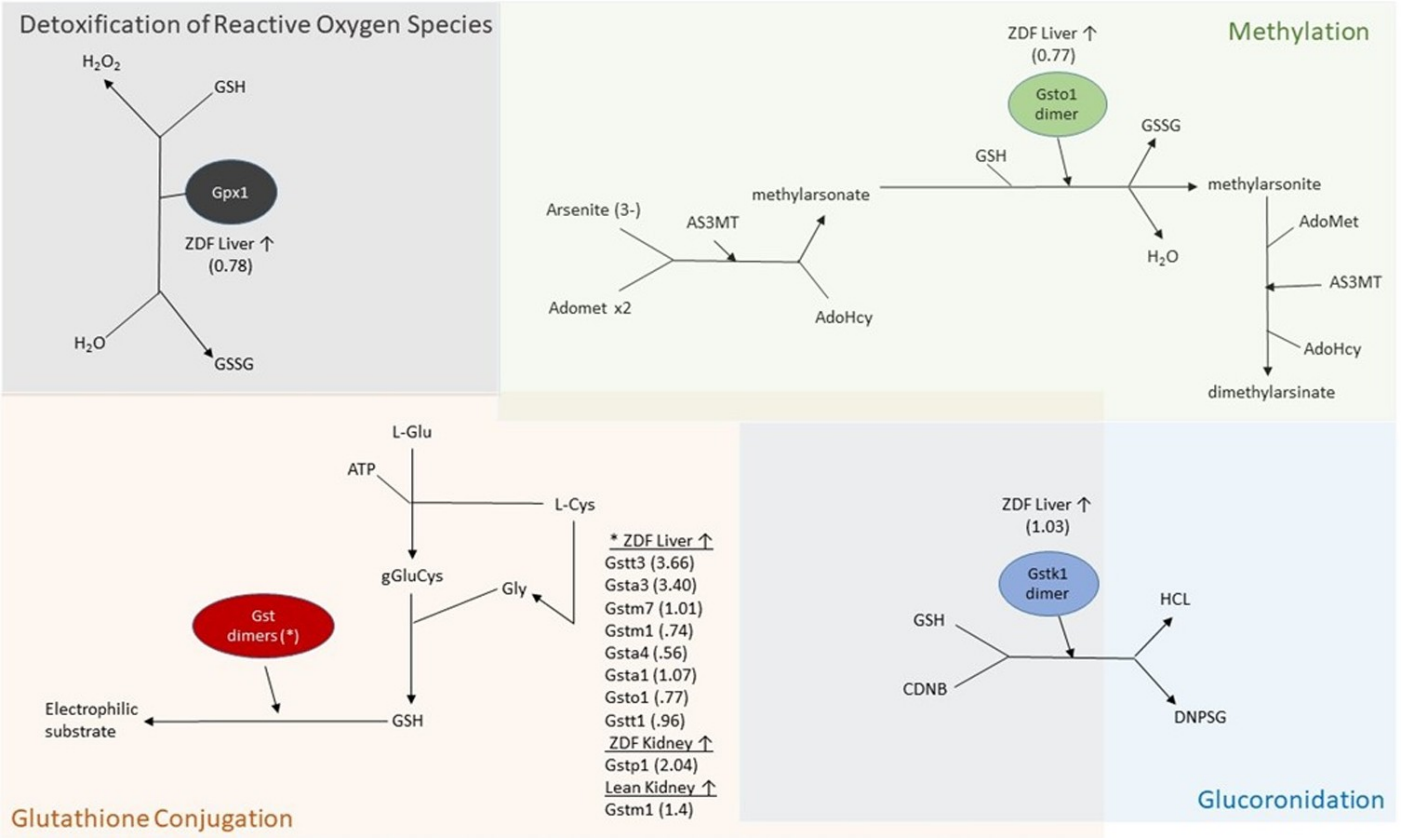
Differentially expressed genes involved in glutathione metabolism. This figure was adapted from D’Eustachio, P., and Jassal, B. from the Reactome [39]. Glutathione metabolism reactions can be categorized into glutathione conjugation, glucuronidation, methylation, or detoxification of reactive oxygen species. All genes are listed within each reaction category followed by their corresponding log2fold change in parentheses for each given tissue. Abbreviations used: ZDF, Zucker Diabetic fatty rat; GSSG, glutathione disulfide; GSH, glutathione; AS3MT, arsenite 3-methyltransferase; AdoMet, S-adenosyl methionine; AdoHcy, S-adenosyl homocysteine; CDNB, 1-chloro-2, 4-dinitrobenzene; DNPSG, S-(2,4-dinitrophenyl)glutathione; glu, glutamate; cys, cysteine; gly, glycine; gGluCys, gamma-glutamyl-L-cysteine; GST, glutathione s-transferase; and GPX, glutathione peroxidase.

To further investigate the specific genes involved in the glutathione metabolism pathways, genes were categorized into the corresponding reactions identified by Reactome.org in **Fig 3.** Glutathione metabolism functions in antioxidant defense, signal transduction, cytokine production, and other cellular processes such as detoxification. The role of GST, GSTK, GSTO dimers, and GPX1 which function in glutathione conjugation, glucuronidation, methylation, and detoxification of reactive oxygen species, respectively, are detailed within **Fig 3**. These reactions within glutathione metabolism are essential for recycling of glutathione disulfide or the conjugation of GSH that can be utilized in redox reactions.

KEGG pathway analysis highlighted that in addition to an upregulation of glutathione metabolism pathways, several of the same gene products mediate metabolism of xenobiotics, a pathway upregulated in our rats fed WE-based diets. Xenobiotic metabolism has previously been shown to be downregulated during insulin dependent T1DM [27], where in this study these pathways were upregulated in response to feeding WE-based diets. These observed effects appear to be tissue specific, as these alterations were the most prominent in ZDF liver, whereas one gene, glutathione s-transferase p (*Gstp1*), was differentially upregulated in the kidney of ZDF rats while glutathione s-transferase mu 1 (*Gstm1*) was upregulated in the lean kidney. These findings support the previous observation that WE consumption affects obese phenotypes differently than a lean phenotype [17], in part, due to the different transcriptomic responses to dietary WE. We previously hypothesized that these differences in response to WE consumption were not due to satiety, because there was increased food intake in the WE group [18]; the present study identifies a potential molecular response to egg partially explaining these previous findings. Other suggested mechanisms that might explain differences between obese and lean genotypes include thermogenesis [40], altered methylation patterns [41], intestinal microbiome alterations [42], and changes in energy expenditure [43]. While there have been numerous studies highlighting differences in the microbiota between obese and lean phenotypes in rats [42] and humans [40], one recent study examining WE consumption concluded that it did not influence the intestinal microbiome in postmenopausal women [44]. Taken together, these observations support the idea that phenotypic alterations during T2DM may depend strongly on obesity status and energy expenditures on a molecular level, potentially in response to changes in the transcriptome.

### qPCR analyses

Finally, we examined the relationship between our qPCR data for several genes to validate the results from the Quantseq analysis. Confirmatory analysis with qPCR demonstrated that across the genes selected, the qPCR data highly correlates with the mRNA Quantseq results (R^2^ = 0.72; **S1 Fig**) indicating strong similarities between these two methods.

### Strengths and limitations

The strengths and limitations of this study should be addressed to better understand how these results fit into the larger context of the current literature. It is estimated that in 2019, people in the United States consumed on average, 5.6 eggs per week [45]. The dose of egg used in this study would equate to roughly 14 eggs per day for a human. While our study demonstrated that consuming a large dose of WE may alter gene expression of various metabolic pathways, particularly during T2DM, this quantity of egg would not be a standard dietary practice in humans. We do recognize that our whole egg dosage was high, but the goal was to examine whether there was a transcriptomic response from consuming dietary whole egg in a T2DM model. It is worth noting that our laboratory has previously reported in ZDF rats that even smaller dosages, such as the human equivalent of <2 eggs/day, significantly reduced weight gain in the ZDF rat and therefore may be effective in identifying oxidative stress outcomes from long-term dietary whole egg consumption [18]. After the examination of the transcriptome following our high WE-based diet, it is warranted to examine these specific genes in a follow-up intervention study. Future studies will focus on titrating down the egg dosages to discern the smallest dosage to elicit similar transcriptomic responses to egg consumption that will be more translatable to human consumption patterns. Overall, our findings are significant as we are the first to report that whole hen egg consumption promotes glutathione metabolism expression during T2DM and alters the transcriptome of multiple tissues using next-generation sequencing. Additionally, we provide evidence supporting the idea that egg consumption modifies endogenous microRNA expression in a tissue-specific manner.

In summary, we examined whether feeding WE modifies expression of microRNAs or gene expression profiles across multiple tissues in a diabetic versus a lean rat model. Across all tissues examined with next generation sequencing, we identified that 9 microRNAs were differentially expressed in response to consuming WE. Additionally, we have shown that these microRNAs were related to tissue-specific changes in gene expression, and that 8 wk of consuming diets high in whole egg modified 583 genes across the PFC, kidney, liver, and adipose tissue. KEGG/GO analyses identified that glutathione metabolism was highly upregulated in response to feeding WE and qPCR results validated the sequencing results. These data suggest that high WE consumption may provide beneficial effects during T2DM by improving glutathione metabolism gene expression across multiple tissues and decreasing gene expression in oxidative stress pathways.

## Materials and methods

The data discussed in this publication have been deposited in NCBI's Gene Expression Omnibus [46] and are accessible through GEO Series accession number GSE157491 (https://www.ncbi.nlm.nih.gov/geo/query/acc.cgi?acc=GSE157491). All protocols used within this study have been made publicly available at protocols.io. Protocols have been zipped into one file and can be accessed at dx.doi.org/10.17504/protocols.io.bjgakjse.

### Animal housing and experimental design

This animal study was approved by the Institutional Animal Care and Use Committee (IACUC) at Iowa State University. All animal care was performed according to Laboratory Animal Resources Guidelines at Iowa State University. Male ZDF (*fa/fa*) rats (*n* = 12) and their lean controls (*fa*/+; *n* = 12) were obtained at 6-wk of age (Charles River, Wilmington, MA). Rats were dual-caged and acclimated for 72 h in conventional cages in a temperature-controlled room (25°C) with a 12-h light-dark cycle. Rats were randomly assigned to an experimental diet **(Table 4)** consisting of either a casein (CAS)-based diet, or a WE-based diet containing dried WE powder (Rose Acre Farms).

**Table 4:** Composition of the CAS and WE based diets fed to lean and ZDF rats for 8 wk^1^.

	CAS	WE
Ingredient (g/kg)		
Casein	200	-
Whole Egg^2^	-	435
Cornstarch	417	365
Corn Oil	183	-
Glucose Monohydrate	150	150
Mineral Mix	35	35
Vitamin Mix	10	10
Choline Bitartrate	2	2
L-methionine	3	3
Biotin (1%)	-	0.4
Macronutrients (% total kcal)^3^		
Protein	17	17
Carbohydrate	48	48
Fat	35	35
Caloric Content	4,715	4,715

^1^All ingredients were purchased from Envigo except for dried whole egg (Rose Acre Farms, Guthrie Center, IA), as well as l-methionine and choline bitartrate (Sigma-Aldrich). Abbreviations used: CAS, casein-based diet, WE, whole egg-based diet.

^2^ Total protein and lipid content provided by 435 g of dried WE was 46% (200 g) and 42% (183 g), respectively.

^3^ To formulate all diets such that protein was provided at 20% (w/w).

Both diets provided 20% protein (w/w) from either vitamin-free CAS or WE powder. To match the diets for total lipid content (18.3%), corn oil was added to the control diet. Both diets were prepared in-house weekly by mixing all ingredients into a powdered form and administered daily in a standard amount for both lean and ZDF rats. For the remainder of the study, rats were fed *ad libitum* for 8 wk and at the end of the experimental period, rats were anesthetized with a dissociative agent combination of ketamine:xylaxine (90:10 mg/kg body weight) via an intraperitoneal injection of 1μL/g body weight. Two methods of animal euthanasia were performed according to the American Veterinary Medical Association guidelines for the Euthanasia of Animals: 2020 edition [47]. Cardiac exsanguination of whole blood on the anesthetized rat was performed and serum was subsequently stored at −80°C for downstream analysis. The second method of exsanguination was the procurement of organs. Following cardiac puncture, tissues were immediately excised, weighed, and snap frozen in liquid nitrogen for storage at −80°C in RNA*Later*.

### RNA extraction and analysis

Tissue samples (20 mg) were rapidly thawed on ice and largeRNA and smallRNA fractions were extracted from the same isolate using the RNA SPLIT Kit (Lexogen) according to the manufacturer’s instructions. Briefly, samples were homogenized in an isolation buffer and phase separated using a phenol/chloroform extraction followed by a spin column-based purification procedure. All samples were aliquoted and stored at −80°C for downstream analysis. Following extraction, sample concentrations for the largeRNA fraction were analyzed using a Qubit 2.0 fluorometer (Thermo Fisher) using the Qubit™ Broad Range RNA Assay Kit. RNA integrity was assessed using the Bioanalyzer 2100 (Agilent Technologies) and samples with low RNA integrity number (RIN) values <5 were discarded and re-extracted. SmallRNA concentrations were measured using a Qubit 2.0 fluorometer (Thermo Fisher) using the Qubit™ microRNA Assay Kit.

### TotalRNA and smallRNA sequencing

Libraries for totalRNA were prepared using an automated protocol according to the manufacturer’s instructions for half reactions on the QuantSeq 3' mRNA-Seq Library Prep Kit (Lexogen) using a MANTIS^®^ Liquid Handler pipetting robot (Formulatrix). All totalRNA samples were multiplexed together across two lanes on an Illumina High-Seq 3000. SmallRNA Libraries were prepared manually using the SmallRNA-Seq Library Prep Kit (Lexogen). Briefly, 100 ng of enriched smallRNA was used as input and 3’ and 5’ adapters were ligated followed by column purifications. Subsequently, the ligation products were reverse transcribed and double stranded cDNA libraries were generated. Finally, individual sample barcodes for multiplexing were introduced via 17 cycles of PCR. All libraries were assessed on the Bioanalyzer 2100 (Agilent) to examine if adapter dimers formed during PCR. All libraries were further prepared using a bead purification module (Lexogen) and pooled into a single sample at 2 nM (20 μL reaction) for sequencing.

### Sequencing quality control and adapter trimming

For both totalRNA and smallRNA samples, the resulting FASTQ files were analyzed using Fast-QC [48] and sequencing adapters were trimmed using on BBDUK [49] with an example of the trimming procedure: bbduk.sh in = reads.fq out = clean.fq maq = 10 ref = /bbmap/resources/adapters.fa. For smallRNA samples, reads were additionally trimmed using the literal flag to remove the Lexogen specific sequence “5’-TGGAATTCTCGGGTGC CAAGGAACTCCAGTCAC– 3’” following similar trimming procedures. Briefly, any read segments that matched Illumina Truseq or Nextera adapters, along with reads containing integrity scores <10 were trimmed out.

### Alignment and read quantification

For totalRNA, reads were mapped to the Ensembl release 94 of the Rattus Norvegicus RNO_6.0 genome using RNA STAR [50]. TotalRNA read counts were generated during the read alignment using the—genecounts function in STAR. For smallRNA samples, reference fasta files from www.RNACentral.org were downloaded for microRNA, piwiRNA, snRNA, nRNA, rRNA, and tRNA. Indexes were generated using Bowtie [50] and alignment was conducted using the smallrnaseq python tool [51]. Read counts for all reference indices and IsomiRs were generated using the smallrnaseq python tool.

### Data filtering and normalization

Following read count generation, Quantseq gene expression data was merged into a single data frame for analysis in R (version 3.6.0). Genes were discarded from the analysis if there were <3 samples without a single read for that given gene. TotalRNA data initially generated read counts for 32,883 genes and over 50% of the trimmed reads from each sample mapped to the RNO_6 version 94 genome. Prior to normalization, remaining gene counts across all four tissues contained between 8,700–12,000 genes for analysis. The microRNA data originally generated read counts for over 350 microRNAs and the formal analysis was conducted on 60–150 targets across each tissue. For totalRNA and smallRNA fractions, all samples were normalized using the Trimmed Mean of M values (TMM) method [52]. Briefly, TMM accounts for variable depth between samples by normalizing them according to the weighted trimmed mean of the log expression ratios across all samples prior to analysis.

### Differential expression analysis

All differential expression analyses were conducted using R (version 3.6.0). Differential expression was conducted using DESeq2 from Bioconductor. DESeq-DataSetFromMatrix generated p-values and Benjamini-Hochberg [53] adjusted *P*-values controlling false discovery rate (FDR) at 5%. Significance was determined at *adj P*<0.05.

### Heatmaps, principal component analysis, and volcano plots

Principal Component Analysis (PCA) was used to visualize sample relatedness across treatments and tissues. Subsequent hierarchical clustering grouped samples according to transcriptomic relatedness, while volcano plots were constructed to visualize samples with absolute log-fold changes >1.5. All figures were generated with MatplotLib in Python version 3.2.0rc1.

### KEGG/GO pathway analysis

Biological pathways for each DEG were generated using the KEGG pathway analysis and GO analysis conducted via the Database for Annotation, Visualization, and Integrated Discovery (DAVID) v6.7 software tool.

### qPCR validation analyses

TotalRNA from each tissue was aliquoted and frozen at -80^○^C, and 2 μg of total RNA was reverse-transcribed into cDNA using the High-Capacity cDNA Reverse Transcription Kit (Applied Biosystems, Catalog # 4368813). cDNA was diluted to 250 ng/μL and qPCR reactions were performed using 250 ng of total cDNA with primers at 300 nM concentration in 10 μL FastStart Sybr Green Master (Roche) according to the manufacturer’s instructions. Briefly, the thermocycling protocol followed a pre-incubation at 95°C for 10 minutes, followed by 45 cycles of 3-Step amplification: 1) denature at 95°C for 20 seconds; 2) anneal and extend at 60°C for 20 seconds; and 3) elongate at 72°C for 20 seconds. All qPCR reactions were conducted in a Roche LightCycler 96 Real-Time PCR System. Primers sequences for qPCR are as follows: Fatty Acid Synthase FWD: GGCGAGTCTATGCCACTATTC, REV: GCTGATACAGAGAACGGATGAG; Indolethylamine N-methyltransferase FWD: CTGGAGAAGGAGACGGTAGAA, REV: CGGGCAACCACGAAGTATAA; Cytochrome P450, family 2, subfamily c, polypeptide 22 FWD: AGAGAGAGAGAGAGAGAGAGAGA, REV: GAGACCCTCTGCATCTCAATAC; 18S Ribosomal Subunit FWD: AAGACGAACCAGAGCGAAAG, REV:TCGGAACTACGACGGTATCT; Cytochrome P450, family 51 FWD: CCTTCCAGTGGTGCTCTTATT, REV: CTAAGCCACTACCCAAAGACTATAC. In all qPCR experiments, 18s RNA expression was used to normalize gene expression within each tissue sample that was processed in triplicate. All data were analyzed using the Livak Delta-Delta CT method [54].

### MicroRNA bioinformatic analysis

All microRNA fastq files were processed using the smallrnaseq [51] package in python. Smallrnaseq automates standard bioinformatic processes for quantification and analysis of small non-coding RNA species such as microRNA quantification and novel microRNA prediction. Briefly, smallrnaseq uses bowtie to align fastq files to user defined reference fasta sequences and all reference sequences were downloaded from www.RNAcentral.org (version 14). Following alignment to the Rattus Norvegicus genome and reference tRNA, rRNA, microRNA, lncRNA, and snRNA files, novel microRNA predictions are conducted using microRNADeep2. Additionally, differential expression was automated using the DEseq2 package in R.

## Supporting information

S1 Fig qPCR correlation with mRNA sequencing. Log fold change comparisons between qPCR and mRNA sequencing of several genes suggesting strong relationship between these two methods.
(TIF)

S1 Table mRNA raw read counts. Raw microRNA counts used in the analysis for comparing dietary treatment groups.
(XLSX)

S2 Table mRNA DeSEQ2 summary statistics. Summary statistics for the mRNA data from the DeSEQ2 analysis in R.
(XLSX)

S3 Table MicroRNA DeSEQ2 summary statistics. Summary statistics for the microRNA data from the DeSEQ2 analysis in R.
(XLSX)

S4 Table Raw microRNA read counts. Raw microRNA read counts used in the analyses.
(XLSX)

S5 Table KEGG/GO analysis. Gene ontology pathways that were upregulated/downregulated for each set of differentially expressed genes within each tissue using the DAVID database.
(XLSX)

## References

[1] Zheng Y, Ley SH, Hu FB. Global aetiology and epidemiology of type 2 diabetes mellitus and its complications. Nature Reviews Endocrinology. Nature Publishing Group; 2018 pp. 88–98. 10.1038/nrendo.2017.151 29219149

[2] Folli F, Corradi D, Fanti P, Davalli A, Paez A, Giaccari A, et al The Role of Oxidative Stress in the Pathogenesis of Type 2 Diabetes Mellitus Micro- and Macrovascular Complications: Avenues for a Mechanistic-Based Therapeutic Approach. Curr Diabetes Rev. 2012;7: 313–324. 10.2174/157339911797415585 21838680

[3] Raza H, John A, Howarth FC. Increased Oxidative Stress and Mitochondrial Dysfunction in Zucker Diabetic Rat Liver and Brain. Cell Physiol Biochem. 2015;35: 1241–1251. 10.1159/000373947 25766534

[4] Sekhar R V., Mckay S V., Patel SG, Guthikonda AP, Reddy VT, Balasubramanyam A, et al Glutathione synthesis is diminished in patients with uncontrolled diabetes and restored by dietary supplementation with cysteine and glycine. Diabetes Care. 2011;34: 162–167. 10.2337/dc10-1006 20929994 PMC3005481

[5] Raza H, John A, Howarth FC. Alterations in glutathione redox metabolism, oxidative stress, and mitochondrial function in the left ventricle of elderly zucker diabetic fatty rat heart. Int J Mol Sci. 2012;13: 16241–16254. 10.3390/ijms131216241 23203193 PMC3546687

[6] Kanikarla-Marie P, Micinski D, Jain SK. Hyperglycemia (high-glucose) decreases l-cysteine and glutathione levels in cultured monocytes and blood of Zucker diabetic rats. Mol Cell Biochem. 2019;459: 151–156. 10.1007/s11010-019-03558-z 31172369

[7] Tessari P. Effects of insulin on whole-body and regional amino acid metabolism. Diabetes Metab Rev. 1994;10: 253–285. 10.1002/dmr.5610100304 7835172

[8] Patel D, Rooney R, Groom S. Gene Expression Profiles for the Zucker Fatty Rat Versus Zucker Diabetic Fatty Rat are Highly Consistent with Those Observed in Human Patients. Available: www.criver.com

[9] Cordero-Herrera I, Martín MÁ, Goya L, Ramos S. Cocoa intake ameliorates hepatic oxidative stress in young Zucker diabetic fatty rats. Food Res Int. 2015;69: 194–201. 10.1016/j.foodres.2014.12.039 25559866

[10] Zou XR, Zhan LR, Chen L, Long QH, Yuan J, Wang L, et al Influence of the klotho/FGF23/egr1 signaling pathway on calciumphosphorus metabolism in diabetic nephropathy and the intervention of shenyuan granules. J Biol Regul Homeost Agents. 2019;33: 1695–1702. 10.23812/19-207-A 31989808

[11] Yousr M, Howell N. Antioxidant and ACE inhibitory bioactive peptides purified from egg yolk proteins. Int J Mol Sci. 2015;16: 29161–29178. 10.3390/ijms161226155 26690134 PMC4691102

[12] Fuller NR, Caterson ID, Sainsbury A, Denyer G, Fong M, Gerofi J, et al The effect of a high-egg diet on cardiovascular risk factors in people with type 2 diabetes: the Diabetes and Egg (DIABEGG) study—a 3-mo randomized controlled trial. Am J Clin Nutr. 2015;101: 705–713. 10.3945/ajcn.114.096925 25833969

[13] Fuller NR, Sainsbury A, Caterson ID, Markovi TP. Egg consumption and human cardio-metabolic health in people with and without diabetes. Nutrients. MDPI AG; 2015 pp. 7399–7420. 10.3390/nu7095344 26404366 PMC4586539

[14] Djoussé L, Khawaja OA, Gaziano JM. Egg consumption and risk of type 2 diabetes: a meta-analysis of prospective studies1. Am J Clin Nutr. 2016;103: 474–480. 10.3945/ajcn.115.119933 26739035

[15] Virtanen JK, Mursu J, Tuomainen T-P, Virtanen HE, Voutilainen S. Egg consumption and risk of incident type 2 diabetes in men: the Kuopio Ischaemic Heart Disease Risk Factor Study. Am J Clin Nutr. 2015;101: 1088–1096. 10.3945/ajcn.114.104109 25832339

[16] Garcés-Rimón M, González C, Uranga JA, López-Miranda V, López-Fandiño R, Miguel M. Pepsin Egg White Hydrolysate Ameliorates Obesity-Related Oxidative Stress, Inflammation and Steatosis in Zucker Fatty Rats. Peterson J, editor. PLoS One. 2016;11: e0151193 10.1371/journal.pone.0151193 26985993 PMC4795625

[17] Saande CJ, Jones SK, Rowling MJ, Schalinske KL. Whole Egg Consumption Exerts a Nephroprotective Effect in an Acute Rodent Model of Type 1 Diabetes. J Agric Food Chem. 2018;66: 866–870. 10.1021/acs.jafc.7b04774 29345464

[18] Saande CJ, Webb JL, Curry PE, Rowling MJ, Schalinske KL. Dietary Whole Egg Reduces Body Weight Gain in a Dose-Dependent Manner in Zucker Diabetic Fatty Rats. J Nutr. 2019;149: 1766–1775. 10.1093/jn/nxz143 31254347

[19] Dhas Y, Mishra N, Banerjee J. Vitamin D Deficiency and Oxidative Stress in Type 2 Diabetic Population of India. Cardiovasc Hematol Agents Med Chem. 2017;14: 82–89. 10.2174/1871525714666160426150233 27114101

[20] Saande CJ, Steffes MA, Webb JL, Valentine RJ, Rowling MJ, Schalinske KL. Whole Egg Consumption Impairs Insulin Sensitivity in a Rat Model of Obesity and Type 2 Diabetes. Curr Dev Nutr. 2019;3 10.1093/cdn/nzz099 31008440 PMC6462456

[21] Pourafshar S, Akhavan NS, George KS, Foley EM, Johnson SA, Keshavarz B, et al Egg consumption may improve factors associated with glycemic control and insulin sensitivity in adults with pre- and type II diabetes. Food Funct. 2018;9: 4469–4479. 10.1039/c8fo00194d 30073224

[22] Dehghan M, Mente A, Rangarajan S, Mohan V, Lear S, Swaminathan S, et al Association of egg intake with blood lipids, cardiovascular disease, and mortality in 177,000 people in 50 countries. Am J Clin Nutr. 2020;111: 795–803. 10.1093/ajcn/nqz348 31965140 PMC7138651

[23] Geiker NRW, Lytken Larsen M, Dyerberg J, Stender S, Astrup A. Egg consumption, cardiovascular diseases and type 2 diabetes. European Journal of Clinical Nutrition. Nature Publishing Group; 2018 pp. 44–56. 10.1038/ejcn.2017.153 28952608

[24] Tran NL, Barraj L, Heilman J, Scrafford C. Egg consumption and cardiovascular disease among diabetic individuals: a systematic review of the literature. Diabetes, Metab Syndr Obes Targets Ther. 2014;7: 121 10.2147/DMSO.S58668 24711708 PMC3969252

[25] Aba P, Igwebuike D, Onah J. Effects of Various Concentrations of Quail Egg Solution on Glycemia and Antioxidant Parameters of Alloxan-induced Diabetic Rats. J Adv Med Pharm Sci. 2016;5: 1–7. 10.9734/jamps/2016/22723

[26] Evans JL, Goldfine ID, Maddux BA, Grodsky GM. Are oxidative stress—Activated signaling pathways mediators of insulin resistance and β-cell dysfunction? Diabetes. American Diabetes Association; 2003 pp. 1–8. 10.2337/diabetes.52.1.1 12502486

[27] Raza H, Ahmed I, John A, Sharma AK. Modulation of xenobiotic metabolism and oxidative stress in chronic streptozotocin-induced diabetic rats fed withMomordica charantia fruit extract. J Biochem Mol Toxicol. 2000;14: 131–139. 10.1002/(sici)1099-0461(2000)14:3<131::aid-jbt2>3.0.co;2-q 10711628

[28] Quigley JD. Effects of Spray-Dried Whole Egg and Biotin in Calf Milk Replacer. J Dairy Sci. American Dairy Science Association; 2002 10.3168/jds.S0022-0302(02)74068-X 11860112

[29] Chen X, Du Y, Boni GF, Liu X, Kuang J, Geng Z. Consuming egg yolk decreases body weight and increases serum HDL and brain expression of TrkB in male SD rats. J Sci Food Agric. 2019;99: 3879–3885. 10.1002/jsfa.9610 30680735

[30] Corbett SW, Stern JS, Keesey RE. Energy expenditure in rats with diet-induced obesity. Am J Clin Nutr. 1986;44: 173–180. 10.1093/ajcn/44.2.173 3728354

[31] Montero D, Tachibana C, Rahr Winther J, Appenzeller-Herzog C. Intracellular glutathione pools are heterogeneously concentrated. Redox Biol. 2013;1: 508–513. 10.1016/j.redox.2013.10.005 24251119 PMC3830055

[32] Shen N, Yu X, Pan FY, Gao X, Xue B, Li CJ. An early response transcription factor, Egr-1, enhances insulin resistance in type 2 diabetes with chronic hyperinsulinism. J Biol Chem. 2011;286: 14508–14515. 10.1074/jbc.M110.190165 21321112 PMC3077649

[33] Garnett KE, Chapman P, Chambers JA, Waddell ID, Boam DSW. Differential gene expression between Zucker Fatty rats ad Zucker Diabetic Fatty rats: A potential role for the immediate-early gene Egr-1 in regulation of beta cell proliferation. J Mol Endocrinol. 2005;35: 13–25. 10.1677/jme.1.01792 16087718

[34] Zhang J, Zhang Y, Sun T, Guo F, Huang S, Chandalia M, et al Dietary obesity-induced Egr-1 in adipocytes facilitates energy storage via suppression of FOXC2. Sci Rep. 2013;3: 1–10. 10.1038/srep01476 23502673 PMC3600596

[35] Li Tiangang, Owsley Erika, Matozel Michelle, Hsu Peter, Chiang John Y.L.. Transgenic expression of CYP7A1 in the liver prevents high fat diet-induced obesity and insulin resistance in mice | The FASEB Journal. Pharmacol Ther. 2010 [cited 18 Jun 2020]. Available: https://www.fasebj.org/doi/abs/10.1096/fasebj.24.1_supplement.570.4 10.1002/hep.23721 PMC3700412 20623580

[36] Gutiérrez-Juárez R, Pocai A, Mulas C, Ono H, Bhanot S, Monia BP, et al Critical role of stearoyl-CoA desaturase—1 (SCD1) in the onset of diet-induced hepatic insulin resistance. J Clin Invest. 2006;116: 1686–1695. 10.1172/JCI26991 16741579 PMC1464900

[37] Mugabo Y, Zhao S, Seifried A, Gezzar S, Al-Mass A, Zhang D, et al Identification of a mammalian glycerol-3-phosphate phosphatase: Role in metabolism and signaling in pancreatic β-cells and hepatocytes. Proc Natl Acad Sci U S A. 2016;113: E430–E439. 10.1073/pnas.1514375113 26755581 PMC4743820

[38] Zhang M, Zhu X. miR-9-5p plays an important role in gestational diabetes mellitus (GDM) progression by targeting HK-2. Int J Clin Exp Med. 2018 Available: www.ijcem.com/ 29874342

[39] Jassal B, Matthews L, Viteri G, Gong C, Lorente P, Fabregat A, et al The reactome pathway knowledgebase. Nucleic Acids Res. 2020;48 10.1093/nar/gkz1031 31691815 PMC7145712

[40] Karst H, Steiniger J, Noack R, Steglich HD. Diet-induced thermogenesis in man: Thermic effects of single proteins, carbohydrates and fats depending on their energy amount. Ann Nutr Metab. 1984;28: 245–252. 10.1159/000176811 6476790

[41] Kvaløy K, Page CM, Holmen TL. Epigenome-wide methylation differences in a group of lean and obese women–A HUNT Study. Sci Rep. 2018;8: 1–9. 10.1038/s41598-017-17765-5 30397228 PMC6218540

[42] Chen J, He X, Huang J. Diet Effects in Gut Microbiome and Obesity. J Food Sci. 2014;79: R442–51. 10.1111/1750-3841.12397 24621052

[43] Corbett SW, Stern JS, Keesey RE. Energy expenditure in rats with diet-induced obesity. Am J Clin Nutr. 1986;44: 173–80. 10.1093/ajcn/44.2.173 3728354

[44] Zhu C, Sawrey-Kubicek L, Bardagjy AS, Houts H, Tang X, Sacchi R, et al Whole egg consumption increases plasma choline and betaine without affecting TMAO levels or gut microbiome in overweight postmenopausal women. Nutr Res. 2020 [cited 27 Apr 2020]. 10.1016/j.nutres.2020.04.002 32464420

[45] M. Shahbandeh, Statistica 2020. Per capita consumption of eggs in the U.S. 2020 | Statista. In: Statistica.com [Internet]. 28 Jan 2020 [cited 7 Aug 2020]. Available: https://www.statista.com/statistics/183678/per-capita-consumption-of-eggs-in-the-us-since-2000/

[46] Edgar R, Domrachev M, Lash AE. Gene Expression Omnibus: NCBI gene expression and hybridization array data repository. Nucleic Acids Res. 2002;30: 207–210. 10.1093/nar/30.1.207 11752295 PMC99122

[47] Leary S, Johnson CL. AVMA GUIDELINES FOR THE EUTHANASIA OF ANIMALS: 2020 EDITION AVMA Guidelines for the Euthanasia of Animals: 2020 Edition* Members of the Panel on Euthanasia AVMA Staff Consultants. 2020.

[48] Andrews S. FASTQC. A quality control tool for high throughput sequence data. 2010 [cited 6 Apr 2020]. Available: https://www.bibsonomy.org/person/1f230a919c34360709aa298734d63dca3/author/0

[49] Bushnell B, Rood J, Singer E. BBMerge–Accurate paired shotgun read merging via overlap. PLoS One. 2017 10.1371/journal.pone.0185056 29073143 PMC5657622

[50] Langmead B, Salzberg SL. Fast gapped-read alignment with Bowtie 2. Nat Methods. 2012;9: 357–359. 10.1038/nmeth.1923 22388286 PMC3322381

[51] Farrell D. smallrnaseq: short non coding RNA-seq analysis with Python. Bioarxiv. 2017; 110585 10.1101/110585

[52] Robinson MD, Oshlack A. A scaling normalization method for differential expression analysis of RNA-seq data. Genome Biol. 2010;11: R25 10.1186/gb-2010-11-3-r25 20196867 PMC2864565

[53] Benjamini, Yoav; Hochberg Y. Controlling the False Discovery Rate—a Practical and Powerful Approach to Multiple Testing. Journal of the Royal Statistical Society Series B-Methodological 1995pdf. J R Stat Soc Ser B. 1995. 10.2307/2346101

[54] Livak KJ, Schmittgen TD. Analysis of relative gene expression data using real-time quantitative PCR and the 2-ΔΔCT method. Methods. 2001;25: 402–408. 10.1006/meth.2001.1262 11846609

