# Supplementary figures and images for: Whole egg consumption increases gene expression within the glutathione pathway in the liver of Zucker Diabetic Fatty rats
Source: PLoS One. 2020 Nov 3;15(11):e0240885. doi: 10.1371/journal.pone.0240885 (PMC7608885; doi:10.1371/journal.pone.0240885)

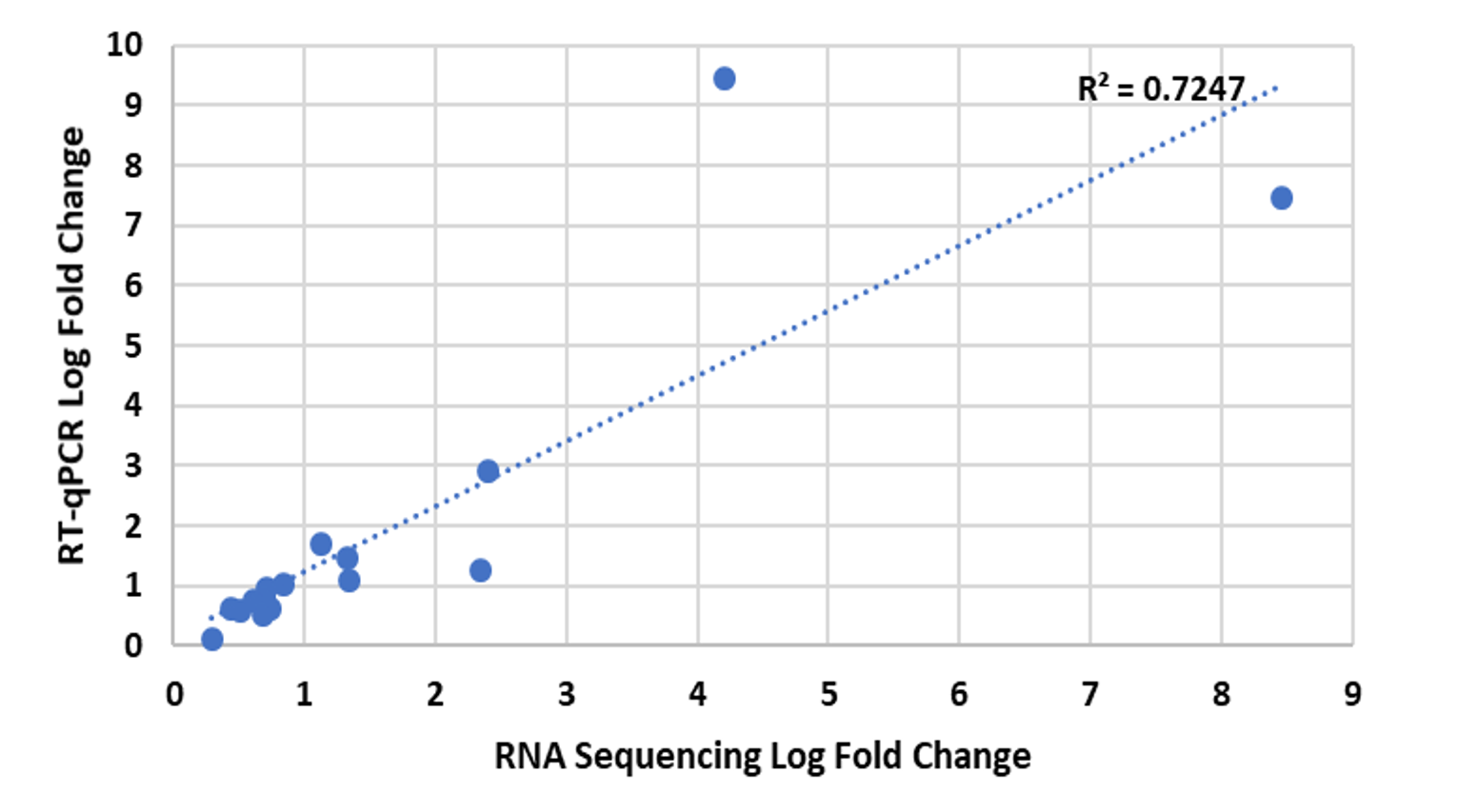

Supplement: S1 Fig — Log fold change comparisons between qPCR and mRNA sequencing of several genes suggesting strong relationship between these two methods. (TIF) [file pone.0240885.s001.tif]
